# Amorphization-enabled decoupling of hydrogen and hydroxide binding on RuSi for efficient alkaline hydrogen evolution

**DOI:** 10.1039/d6sc05983j

**Published:** 2026-09-28

**Authors:** Weizheng Cai, Haotian Guan, Chuanlong Liu, Yangfan Lu, Jiazhen Wu

**Affiliations:** a Department of Materials Science and Engineering, Southern University of Science and Technology Shenzhen 518055 China wujz@sustech.edu.cn; b School of Flexible Electronics, Sun Yat-sen University Shenzhen 518107 China; c College of Materials Science and Engineering, National Engineering Research Center for Mg Alloys, National Key Laboratory of Advanced Casting Technologies, National Innovation Center for Industry-Education Integration of Energy Storage Technology, Chongqing University Chongqing 400045 China yangfanlu@cqu.edu.cn; d Chongqing Institute of New Energy Storage Materials and Equipment Chongqing 401135 China; e College of Materials Science and Engineering, Chongqing University of Technology Chongqing 400054 China htguan@cqut.edu.cn; f Institute of Innovative Materials, Southern University of Science and Technology Shenzhen 518055 China; g State Key Laboratory of Quantum Functional Materials, Southern University of Science and Technology Shenzhen 518055 China

## Abstract

Catalyst optimization for the alkaline hydrogen evolution reaction (HER) is fundamentally constrained by scaling relations between hydrogen binding energy (HBE) and hydroxide binding energy (OHBE), yet rational design strategies capable of synergistically optimizing both descriptors have remained experimentally elusive. Herein, we demonstrate that independent regulation of HBE and OHBE dramatically accelerates alkaline HER kinetics by employing a structurally flexible amorphous RuSi (a-RuSi) as a dual-site model catalyst. Through annealing-induced evolution of structural order and local Ru–Si coordination, the adsorption energetics of H* and OH* are decoupled, which breaks the conventional scaling relationship and shifts the catalytic activity maximum to a higher-performance regime. The disordered structure generates diverse Ru^*δ*−^–Si^*δ*+^ Lewis acid–base pairs *via* ligand-to-metal charge transfer, enabling synergistic H_2_O activation and balanced intermediate binding. This yields an ultralow overpotential of 7.85 ± 0.35 mV at 10 mA cm^−2^ with remarkable durability over 1000 h at 1 A cm^−2^ in 1 M KOH. Critically, this work constructs an experimental 3D activity-HBE–OHBE descriptor map for the RuSi catalyst series, highlighting the importance of balanced H and OH binding in bifunctional alkaline HER catalysis.

## Abstract 2

Disordered Ru–Si dual sites decouple H and OH binding, opening a higher-activity HBE–OHBE regime for alkaline HER and delivering 7.85 mV at 10 mA cm^−2^ with >1000 h durability at 1 A cm^−2^.

## Graphical abstract

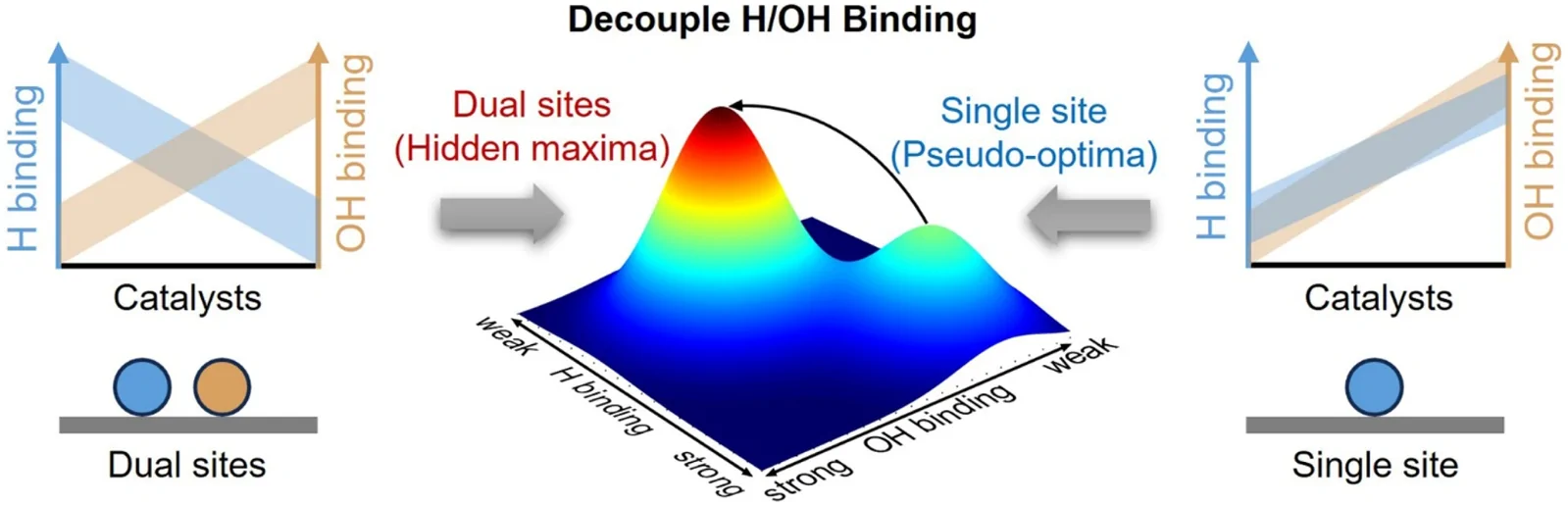

## Introduction

The pursuit of net-zero emissions necessitates scalable, low-carbon hydrogen production technologies. Alkaline water electrolysis, compatible with renewable energy systems and earth-abundant catalysts, presents a promising route.^1–4^ In alkaline media, the hydrogen evolution reaction (HER) mechanism typically involves the initial Volmer step (H_2_O + e^−^ → H* + OH^−^), followed by hydrogen recombination through either the Tafel (H* + H* → H_2_) or Heyrovsky (H_2_O + H* + e^−^ → H_2_ + OH^−^) pathway.^5^ Early studies predominantly associated HER activity with hydrogen binding energy (HBE). This yielded a volcano relationship between catalytic efficiency and HBE, underscoring the necessity of optimizing H* adsorption through electronic structure engineering.^6–8^ However, this HBE-centric paradigm fails to account for the marked performance gap of Pt catalysts between alkaline and acidic conditions, highlighting the crucial role of hydroxide binding energy (OHBE).^9,10^ Thus, balancing HBE and OHBE is essential, yet this is highly challenging because of the scaling relations between H* and OH* adsorption energies (Fig. 1).^11^ These electronic structure correlations, stemming from synchronous valence electron interactions at metal surfaces, inherently limit conventional single-phase catalysts from independently tuning these parameters.

**Fig. 1:**
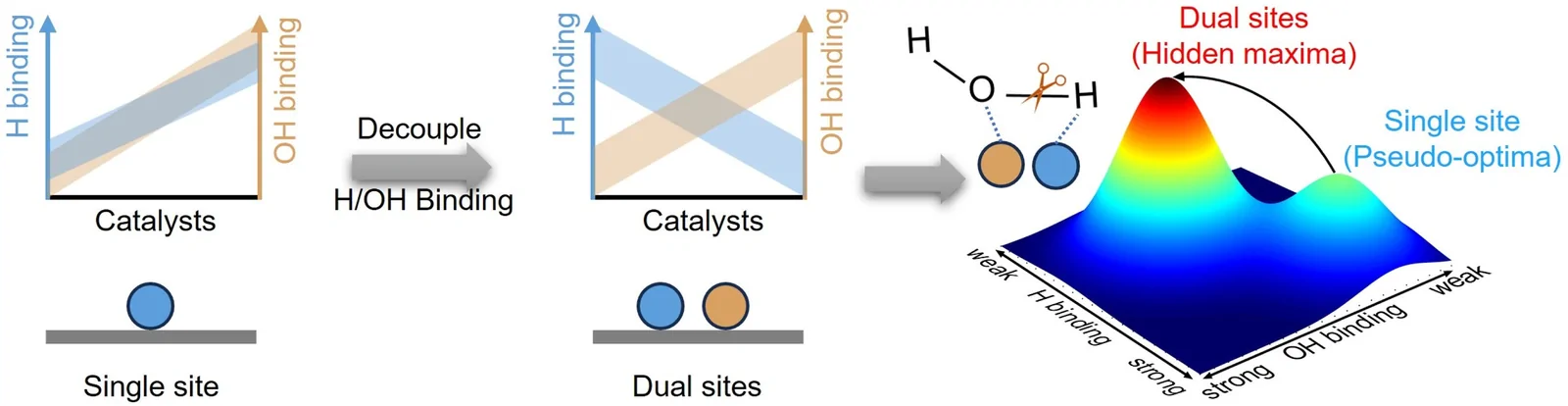
Schematic illustrating how dual-site catalysts decouple H and OH binding, inducing a volcano shift and performance enhancement in the 3D activity-HBE–OHBE volcano plot.

Bifunctional catalysts offer a promising strategy to decouple H* and OH* adsorption.^12^ For instance, enhanced HER kinetics have been demonstrated in Pt/Ni(OH)_2_ heterostructures, where Pt sites favor H* adsorption while Ni(OH)_2_ stabilizes OH*.^13^ The bifunctional approach was further supported by reactivity trends (Ni > Co > Fe > Mn) correlated with OH_ad_–TM (TM: transition metals) interaction strengths in TM(OH)_2_/Pt systems (TM = Ni, Co, Fe, Mn).^14^ Later, this understanding was refined, showing that Pt HER activity exhibits a volcano-shaped dependence on OHBE at fixed HBE, with excessive OH adsorption impeding desorption kinetics.^15^ Similar strategies have been extended to various TM/TMO_*x*_ heterostructures,^16–22^ such as Pt–Ru/RuO_2_ (ref. 16) and Ru/CrO_*x*_.^18^ More recent studies have further demonstrated the importance of dual-active-site design in alkaline HER, including stabilization of water-dissociation sites under high-current operation, porous bimetallic alloys with separated water-activation and hydrogen-adsorption sites, and heterostructures that regulate hydroxyl adsorption/desorption kinetics. These advances highlight that alkaline HER requires coordinated optimization of H_2_O activation, H* adsorption and OH* removal, rather than optimization of a single descriptor alone.^23–27^ However, these catalysts often overlook HBE optimization and are limited by the poor electrical conductivity of TM(OH)_*x*_.^28^ Therefore, despite recognizing HBE and OHBE as key descriptors for catalyst performance, most previous studies have focused on either 2D activity-HBE or activity-OHBE volcano plots, leaving an experimental 3D activity-HBE–OHBE volcano plot elusive.

Subsequently, the bifunctional concept has been more generally extended to multi-site catalytic systems featuring Lewis acid–base pairs (TM–X; TM = Ru, Ir, Pt, *etc.*; X = Si, S, P, *etc.*).^29–33^ This synergy promotes H–OH bond cleavage by enabling selective adsorption of H and OH species at Lewis base (TM) and acid (X) sites, respectively. As the active moieties can be readily adjusted, these catalysts provide a broad exploration space for systematically tuning HBE and OHBE. The Ru–Si dual-site system, featuring a highly flexible structure, has particularly drawn attention as a platform for analyzing structure–performance relationships.^32,33^ The changes in structural order may enable modifications of electronic structures through altered geometric configurations and ligand effects of Ru and Si sites. However, most reported dual-site systems rely on heterointerface construction or compositional alloying, whereas amorphization-enabled regulation of local coordination environments remains less explored. Because annealing can simultaneously alter structural order and surface Si distribution, Ru–Si intermetallics provide an opportunity to examine how local Ru–Si coordination environments regulate HBE and OHBE. This offers an opportunity to experimentally map the coupled dependence of HER activity on HBE and OHBE within the RuSi catalyst series.

Here, we demonstrate that H* and OH* binding can be decoupled on amorphous RuSi (a-RuSi), with HBE and OHBE independently optimized through structural tuning. We experimentally establish a 3D activity-HBE–OHBE descriptor map for the RuSi catalyst series, revealing a hidden and elevated activity peak relative to single-site catalysts (Fig. 1). The optimized catalyst achieves an ultralow overpotential of 7.85 ± 0.35 mV at 10 mA cm^−2^ and a Tafel slope of 29.3 ± 0.42 mV dec^−1^ in 1 M KOH, alongside exceptional stability for 1050 h at industrial-scale current density (1 A cm^−2^). In a-RuSi, Ru^*δ*−^–Si^*δ*+^ Lewis acid–base pairs are formed through electron transfer from Si to Ru driven by Ru's higher electronegativity. These pairs selectively interact with H* and OH*, promoting water dissociation. By modulating the local Ru–Si coordination environment through thermal annealing, the electron distribution (and thus Lewis acidity/basicity) within these pairs can be tuned, enabling adjustable HBE and OHBE. This study establishes an experimental 3D activity-HBE–OHBE descriptor map that reveals the coupled influence of HBE and OHBE on HER activity within the RuSi catalyst series, providing a framework for regulating multiple adsorbate interactions in bifunctional catalysts.

## Results and discussion

### Materials preparation and characterization

The a-RuSi catalyst was synthesized *via* sequential chemical etching of the intermetallic electride precursor LaRuSi.^32,34^ X-ray diffraction (XRD) patterns of the final etched product (Fig. S1) displayed only broad diffraction features, indicating the loss of long-range crystalline order and the formation of a highly disordered Ru–Si structure. Scanning electron microscopy (SEM) with energy-dispersive X-ray spectroscopy (EDS) mapping (Fig. S2) showed uniform distribution of La, Ru, and Si in the precursor. While acid treatment almost completely removed La, it exerted little influence on the Si content. Subsequent alkaline etching removed most of the Si, reducing the SEM-EDS-derived average Si content to ∼1 at%. This value reflects an interaction-volume-averaged composition rather than a surface-specific Si concentration. This remaining silicon plays a key role in stabilizing the disordered Ru–Si matrix structure, as confirmed by subsequent analyses. High-resolution transmission electron microscopy (HRTEM) was used to track the structural evolution during the etching process. Acid treatment yielded a metastable Ru/Si heterostructure with partial crystallinity (Fig. S3). In contrast, alkaline etching generated highly disordered a-RuSi nanosheets, in which extended lattice fringes or well-defined crystalline Ru domains were not observed by HRTEM/AC-TEM. Additional HAADF-STEM images (Fig. S4) collected from different regions further show disordered atomic contrast without extended lattice fringes or well-defined ∼3 nm crystalline Ru domains. Together with the XRD, HRTEM and FFT results, these observations support a predominantly amorphous/highly disordered structure, although very short-range ordered motifs cannot be completely excluded. This systematic progression from an ordered intermetallic precursor to a heterostructure intermediate and a highly disordered Ru–Si structure suggests that the residual Si atoms kinetically hinder Ru lattice reorganization, thereby trapping the system in a disordered state (Fig. S3–5).

To tune the degree of structural disorder, a-RuSi was annealed at temperatures ranging from 200 to 700 °C. XRD patterns (Fig. 2a) displayed progressive sharpening of Ru-related diffraction peaks (PDF #00-06-0663), with crystallite size calculated using the Scherrer equation (Fig. 2b). Below 400 °C, only very small Ru-related ordered domains were detected, indicating that the highly disordered Ru–Si structure is thermally stable in this temperature range. Above this threshold, rapid grain growth (>20 nm) indicated substantial crystallization, consistent with aberration-corrected TEM (AC-TEM) observations (Fig. 2c–f). Pristine a-RuSi showed diffuse diffraction rings in fast Fourier transform (FFT) patterns (Fig. 2c), whereas annealing at 400 °C induced lattice fringes (Fig. 2d), evolving into single-crystalline Ru domains at 700 °C (Fig. 2e and f).

**Fig. 2:**
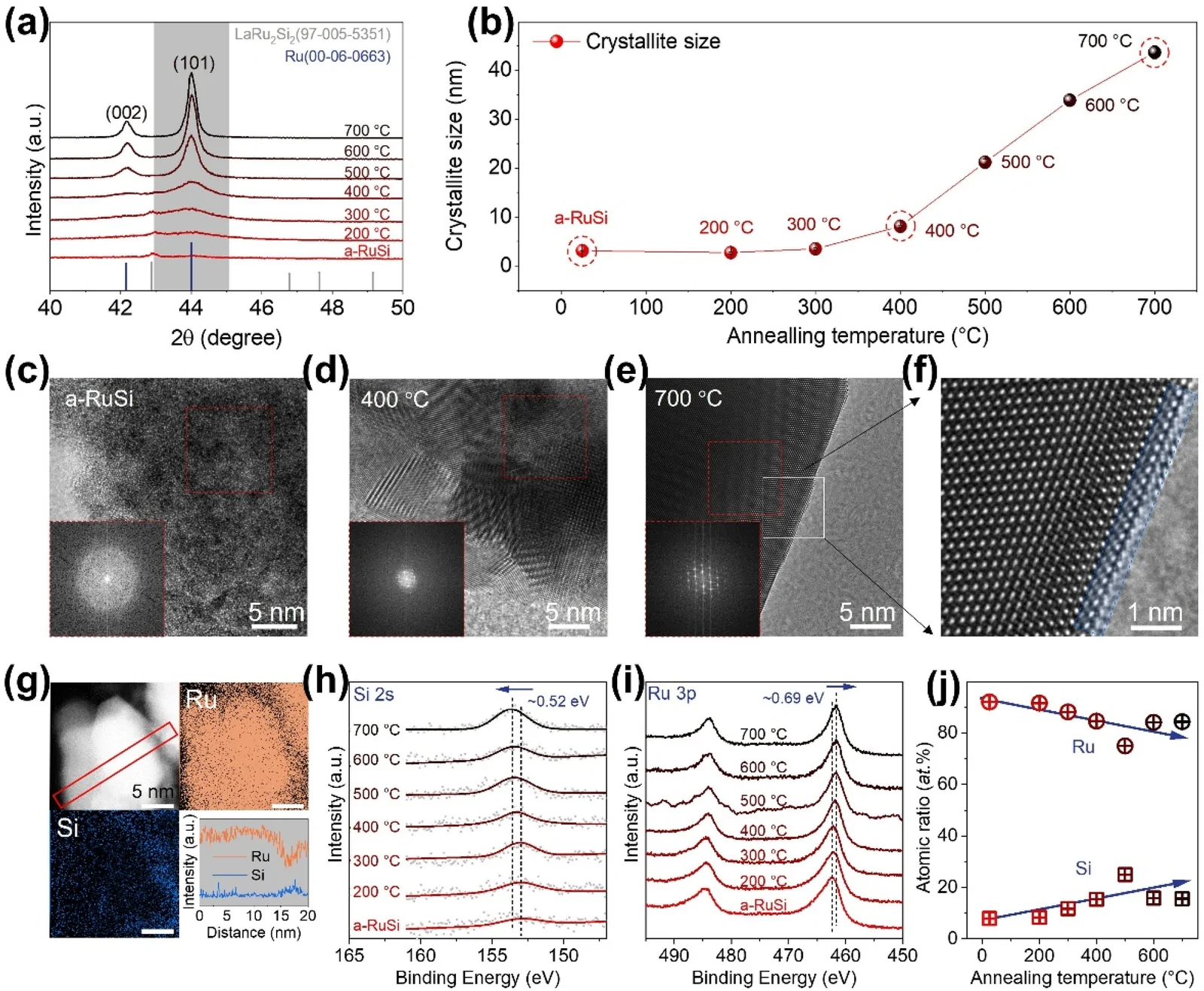
Catalysts preparation and characterization. (a) XRD patterns illustrating the progressive structural ordering and growth of Ru-related crystalline domains during annealing. (b) Crystallite size distribution calculated using the Scherrer equation. (c–e) HRTEM images of pristine a-RuSi and samples annealed at 400 °C and 700 °C, respectively, with corresponding FFT patterns (insets, red-boxed regions). (f) Magnified view of the crystalline lattice in (e). (g) EDS line scans and elemental mapping for the 700 °C-annealed sample. (h and i) High-resolution XPS spectra of Si 2s and Ru 3p for samples annealed from 200 °C to 700 °C (bottom to top). (j) Surface atomic ratios from quantitative XPS analysis.

Strikingly, AC-TEM data of the 700 °C-annealed sample (Fig. 2f) revealed unusual lattice fringe distortions in the outermost ∼3 atomic layers of crystalline Ru, markedly deviating from the bulk lattice's well-defined periodicity. EDS line scans (Fig. 2g) and elemental mapping revealed that this is associated with surface-segregated Ru_*x*_Si_*y*_, identifying a Si-rich surface compared to a-RuSi (Fig. S6). We attribute this effect to Si expulsion during crystallization of the Ru-rich matrix, as collectively evidenced by the correlated structural and chemical data.

X-ray photoelectron spectroscopy (XPS) analysis further quantified electronic interactions during phase evolution. An upshift of 0.53 eV in the Si 2s binding energy (Fig. 2h) and a concurrent downshift of 0.69 eV in the Ru 3p levels (Fig. 2i) indicated charge redistribution between Ru and surface-enriched Si. The increasing Si/Ru atomic ratio with annealing temperature (Fig. 2j) corroborated the formation of Ru_*x*_Si_*y*_ phases, consistent with AC-TEM observations. To verify Si surface segregation during annealing, we computationally compared the structural stability of internal-Si and surface-Si configurations (Fig. S7). The results showed that surface-Si configuration is 1.44 eV more stable than that of the internal-Si structure. Collectively, these findings establish that residual Si not only stabilizes the highly disordered Ru–Si structure but also modulates interfacial electronic states *via* the formation of Ru^*δ*−^–Si^*δ*+^ Lewis acid–base pairs, which are crucial for catalytic performance. It should be noted that thermal annealing changes both the degree of structural disorder and the surface Si distribution. Therefore, the annealing series should not be interpreted as a single-variable crystallinity control, but rather as a systematic modulation of local Ru–Si coordination environments, including structural ordering, Si redistribution, and Ru_*x*_Si_*y*_ surface-motif formation.

### Electrocatalytic performance

The HER activities of a-RuSi and its annealed derivatives were systematically evaluated in 1 M KOH using a three-electrode configuration. Linear sweep voltammetry (LSV) curves (Fig. 3a) showed that a-RuSi outperformed all crystalline counterparts. Overpotentials at 10 mA cm^−2^, averaged from three independent measurements, are compared in Fig. 3d and Table S1. Samples annealed below 400 °C retained similar activity (Δ*η* < 3 mV), whereas further annealing above this threshold, accompanied by substantial crystallization and Si redistribution, markedly increased overpotentials, exceeding 100 mV for fully crystalline variants (c-RuSi). Notably, the Brunauer–Emmett–Teller (BET) surface area decreased monotonically with increasing annealing temperature until 600 °C before stabilizing (Fig. 3b). Because BET surface area does not directly represent the electrochemically active surface area, this trend alone cannot establish whether the activity evolution originates from changes in the number of accessible sites or from intrinsic catalytic properties. Tafel slopes (Fig. 3c and e) further elucidated the reaction kinetics: samples annealed below 500 °C displayed slopes near 30 mV dec^−1^, consistent with a rate-determining Tafel step (2H* → H_2_);^35^ in contrast, highly crystalline catalysts (>500 °C) exhibited slopes > 80 mV dec^−1^, signifying a shift to Volmer-step limitation (H_2_O + e^−^ → H* + OH^−^) and impaired water dissociation. Overall, a-RuSi surpassed reported Ru-based catalysts for alkaline HER, achieving an ultralow overpotential of 7.85 ± 0.35 mV at 10 mA cm^−2^ and a Tafel slope of 29.3 ± 0.42 mV dec^−1^ (Fig. 3f and Table S1). Such a kinetic transition indicates that the local Ru–Si active motifs, rather than surface Si content alone, determine the intrinsic HER behavior.

**Fig. 3:**
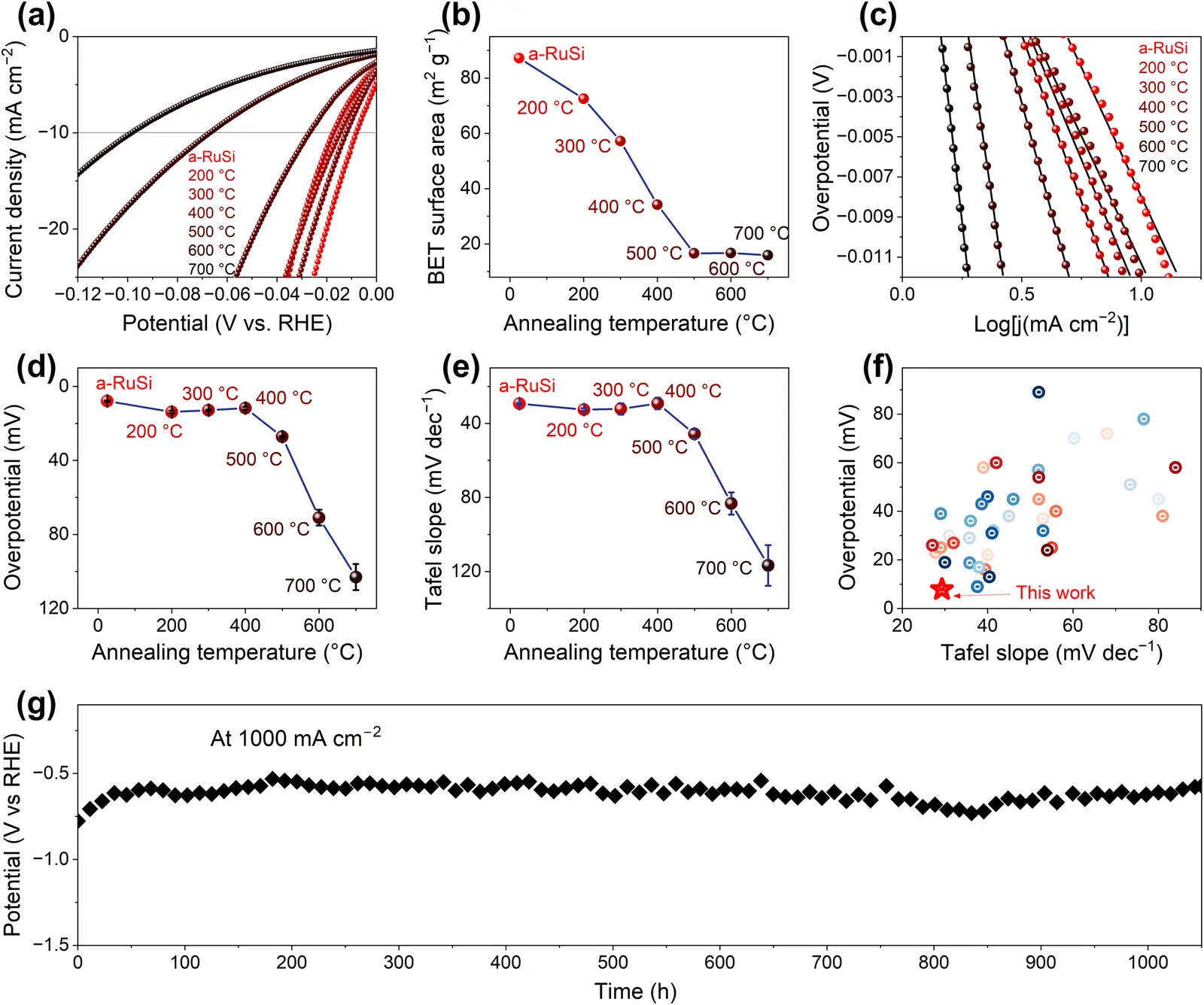
Electrocatalytic performance. (a) Linear sweep voltammetry (LSV) curves of pristine a-RuSi and its annealed derivatives in 1 M KOH at a scan rate of 10 mV s^−1^. (b) Brunauer–Emmett–Teller (BET) surface area analysis of a-RuSi annealed at various temperatures. (c) Corresponding Tafel plots of a-RuSi annealed at various temperatures. (d) Overpotentials at 10 mA cm^−2^, derived from LSV curves, for a-RuSi samples annealed at various temperatures. (e) Corresponding Tafel slopes extracted from the LSV curves in (c). (f) Comparison of overpotentials and Tafel slopes for various alkaline HER catalysts (details in Table S1). (g) Chronopotentiometric stability at a constant current density of 1 A cm^−2^ for up to 1050 h.

Beyond superior activity, a-RuSi's operational robustness at an industrially relevant current density should be highlighted.^36^ The catalyst, drop-cast onto carbon paper with a geometric area of 0.25 cm^2^, was tested by chronopotentiometry at 1 A cm^−2^ in 1 M KOH using a conventional static H-cell configuration. The complete raw potential-time curve is provided in Fig. S8a, and the detailed testing conditions, including electrolyte volume, water-replenishment procedure, and temperature conditions, are described in the SI. The catalyst maintained >95% of its initial performance during chronopotentiometry for over 1050 h without appreciable degradation (Fig. 3g and S8a). Post-stability SEM imaging revealed preserved morphology and chemical composition, while XRD showed no appreciable structural ordering or phase segregation (Fig. S8b and c), demonstrating substantial structural and operational stability under the specified high-current H-cell conditions.

### Analysis of HBE and OHBE

To probe surface hydrogen intermediate properties, hydrogen underpotential deposition (H-UPD)^19,37^ was analyzed *via* cyclic voltammetry (Fig. 4a). The hydrogen desorption peak potential (*E*_H_des__), a key descriptor for balancing Volmer–Heyrovsky/Tafel steps in traditional HBE theory, was determined for various catalysts. The *E*_H_des__ of a-RuSi (0.19 V *vs.* reversible hydrogen electrode (RHE)) shifted anodically by 70 mV relative to crystalline counterparts (0.12 V *vs.* RHE; Fig. 4b), indicating stronger HBE in a-RuSi. *E*_H_des__ correlated negatively with annealing temperature, indicating that the annealing-induced evolution of the local Ru–Si environment is accompanied by weaker H* adsorption (Fig. 4b). Hydrogen desorption kinetics (H* + OH^−^ → H_2_O + e^−^) were further examined through scan-rate-dependent voltammetry^38^ (Figs. S9 and S10). For crystalline catalysts, peak currents scaled linearly with scan rate (*ν*) (log(*I*_p_) *vs.* log(*ν*), slope *α* ≈ 1), a characteristic of surface-limited kinetics. In contrast, a-RuSi exhibited diffusion-controlled behavior (*α* ≈ 0.5, *I*_p_ ∝ *ν*^1/2^), implying rapid H* replenishment *via* its disordered atomic matrix. This kinetic shift suggests that the highly disordered Ru–Si structure facilitates both water dissociation (Volmer step) and proton transport. To verify the assignment of the H-UPD-related feature, CO stripping experiments were performed as an orthogonal surface-blocking probe (Fig. S11). After CO adsorption, the low-potential surface adsorption features were suppressed, while characteristic CO oxidation/desorption signals appeared at higher potentials. These results support that the selected *E*_H_des__ mainly reflects surface H* adsorption/desorption on accessible catalytic sites rather than pure double-layer charging. Therefore, *E*_H_des__ is used here as a relative experimental descriptor for comparing the H* adsorption trend within the RuSi catalyst series. A volcano plot correlating *E*_H_des__ with HER activity (Fig. 4c and S12) positioned Pt at the apex for single-phase metal catalysts (black line), aligning with conventional HBE theory. However, a-RuSi and its annealed derivatives formed a distinct volcano, with a-RuSi exhibiting superior activity despite weaker H* binding than Pt. This highlights the advantages of dual-site catalysts over single-site ones for alkaline HER, emphasizing the role of OHBE alongside HBE in water dissociation kinetics.

**Fig. 4:**
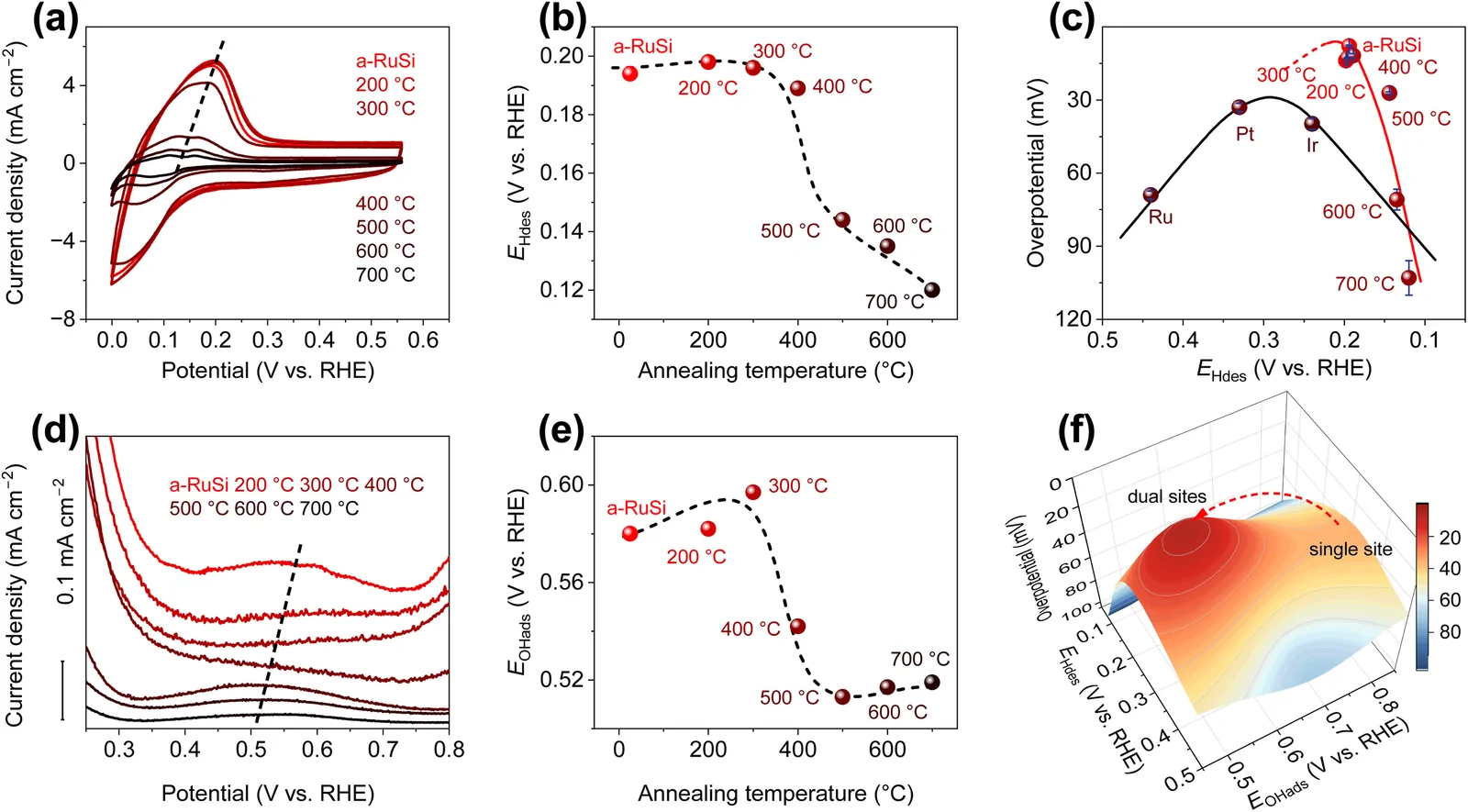
Analysis of HBE and OHBE. (a) Cyclic voltammetry curves at 25 mV s^−1^ in Ar-saturated 0.1 M KOH, with dashed lines indicating characteristic redox peaks. (b) HBE estimated from hydrogen desorption peak potentials (*E*_H_des__) in (a) for each catalyst, showing variation with annealing conditions (higher temperatures yield lower *E*_H_des__). (c) Activity volcano plot correlating *E*_H_des__ with alkaline HER activity. (d) OH* adsorption curves at 25 mV s^−1^ in Ar-saturated 0.1 M KOH, with dashed lines indicating characteristic redox peaks. (e) OHBE estimated from hydroxide adsorption peak potentials (*E*_OH_ads__) in (d) for each catalyst. (f) Experimental 3D activity-HBE–OHBE descriptor map showing dual dependence of HER activity on *E*_H_des__ and *E*_OH_ads__ for the RuSi catalyst series. The interpolated surface is used as a visual guide to illustrate the activity trend.

Subsequently, electrochemical OH* adsorption experiments^39^ were conducted (Fig. 4d, S13 and S14). The OH* adsorption peak potentials (*E*_OH_ads__), which is an indicator of OHBE, are summarized in Fig. 4e for a-RuSi and derivatives; lower potentials indicate stronger OHBE. These potentials correlated negatively with annealing temperature, indicating that the annealing-induced evolution of the local Ru–Si environment is accompanied by stronger OH* adsorption (Fig. 4e). Because the OH* adsorption features are relatively broad, *E*_OH_ads__ was extracted using a consistent baseline-correction procedure for all catalysts and is treated as a semi-quantitative descriptor of relative OH* adsorption tendency rather than an absolute OH* adsorption free energy. To further validate this trend, CO stripping measurements were conducted and the CO* desorption/oxidation onset potential, *E*_CO_des__, was extracted from the tangent intersection of the rising oxidation feature (Fig. S11). As shown in Fig. S15, *E*_CO_des__ follows the same overall trend as *E*_OH_ads__. Since CO* oxidation/desorption in alkaline media requires surface OH* species, this agreement provides independent support for the relative OH* adsorption trend obtained from hydroxide adsorption measurements. Although enhanced OHBE is often linked to improved alkaline HER, the inferior performance of c-RuSi here underscores that excessively strong OH* binding can impede activity. The activity volcano plot relating *E*_OH_ads__ to HER performance (Fig. S16) also shows a shifted peak relative to conventional single-site catalysts, illustrating gains from optimized bifunctional sites. To encapsulate this dual dependency, HER activity was correlated with both *E*_H_des__ and *E*_OH_ads__, yielding an experimental 3D activity-HBE–OHBE descriptor map for the RuSi catalyst series (Fig. 4f). The raw experimental data points are explicitly marked on the interpolated surface, and an additional transparent representation is provided in Fig. S17 for clarity. Within the experimentally sampled descriptor space, the highest activity is observed for a-RuSi, where a favorable balance between HBE and OHBE is achieved. A lower-activity region at weaker OHBE is associated with conventional single-site catalysts, consistent with the constraints imposed by conventional scaling relationships. Although the precise curvature and local maxima of the interpolated surface should not be interpreted quantitatively, the experimental data support that dual-site RuSi catalysts can access a higher-activity HBE–OHBE regime than conventional single-site catalysts. This shift in the accessible activity landscape highlights the importance of independently regulating H* and OH* binding in rational bifunctional catalyst design.

### *Operando* mechanistic investigations

The potential-dependent evolution of interfacial processes during HER was probed using *operando* EIS. Bode phase diagrams (Fig. 5a) and Nyquist plots (Fig. 5b and c) revealed distinct kinetic regimes across the applied potential range (−0.09 to 0.05 V *vs.* RHE). At potentials above 0 V *vs.* RHE, a sharp phase angle attenuation in the low-frequency region (0.1–1 Hz; blue area) corresponds to H-UPD, implicating the Volmer step (H* formation) as dominant.^40^ This aligns with the H-UPD voltammetry in Fig. 4a. The gradual phase angle decrease in the mid-frequency region (around 10^1^–10^3^ Hz; grey area) reflects the sluggish Heyrovsky step.^40^ The high-frequency response (10^4^–10^5^ Hz; red area) arises from electron transfer and charging of the electrical double-layer capacitance (DLC).^41–43^

**Fig. 5:**
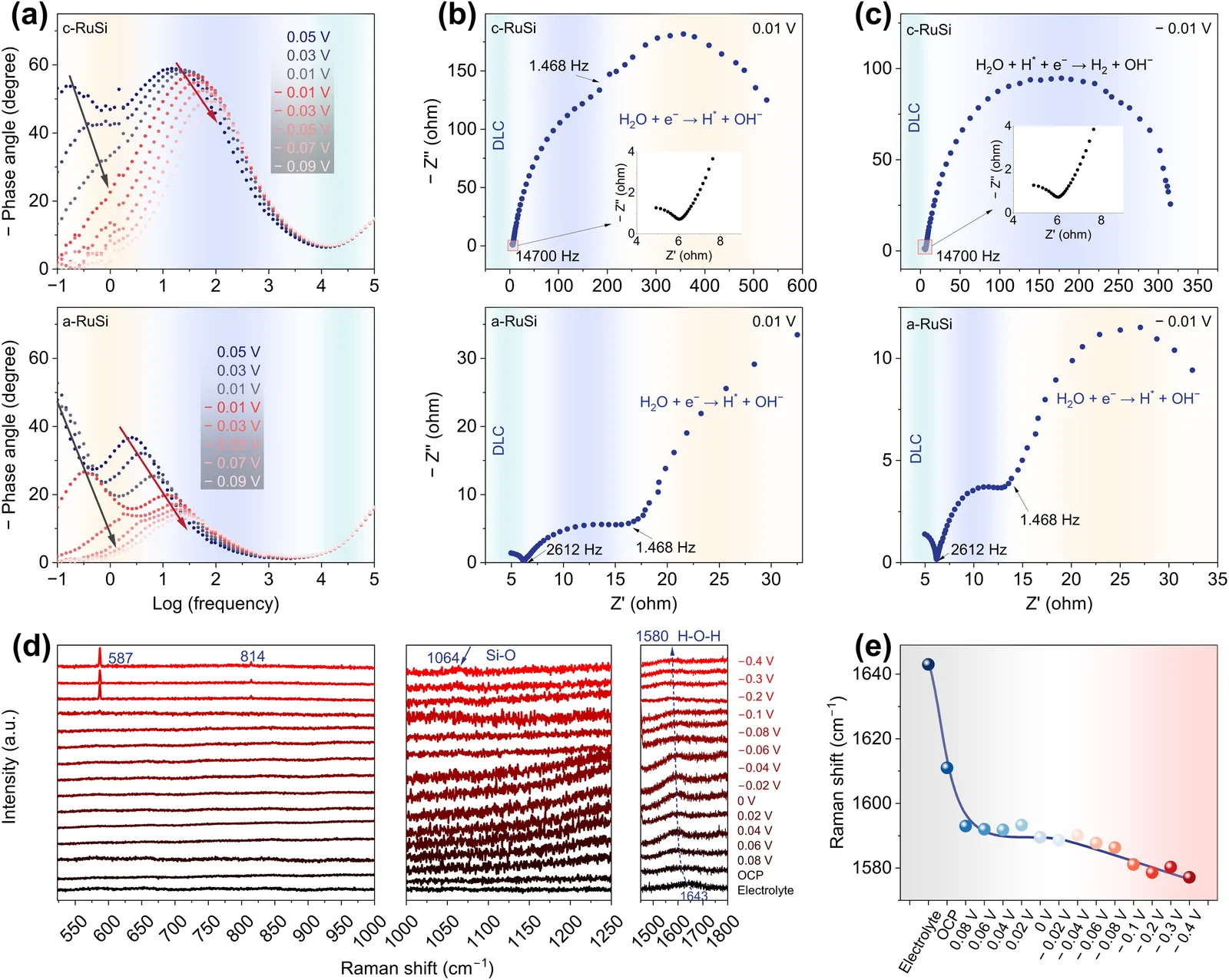
*Operando* mechanistic investigations. (a) Bode phase angle plots for a-RuSi and c-RuSi (annealed at 700 °C) in 1 M KOH. (b and c) Nyquist plots at ± 0.01 V *vs.* RHE. DLC refers to the electrical double-layer capacitance. (d) Potential-dependent *operando* Raman spectra of a-RuSi in 1 M KOH under applied bias *versus* RHE, tracking reaction intermediates. (e) Potential-dependent changes in the H–O–H bending mode, correlating interfacial water restructuring with applied bias *versus* RHE.

Despite these shared features, marked kinetic differences emerged between c-RuSi and a-RuSi. For c-RuSi, with Volmer-limited kinetics (Tafel slope: 116.8 ± 10.9 mV dec^−1^), increasing overpotential led to rapid collapse of the low-frequency phase angle, vanishing below 0 V *vs.* RHE (Fig. 5a–c). This indicates low H* coverage. The mid-frequency regime (10–100 Hz) exhibited weak phase attenuation (Fig. 5a and c), indicative of hindered water dissociation processes associated with the reaction kinetics. In contrast, a-RuSi exhibited resolved low- and mid-frequency signals below 0 V *vs.* RHE (Fig. 5a and c), signifying concurrent Volmer and Heyrovsky pathways. However, its experimental Tafel slope of 29.3 ± 0.42 mV dec^−1^ suggests that the rate-determining step is associated with the Heyrovsky or Tafel process rather than the Volmer step.^44^ As the chemical Tafel step (2H* → H_2_) is not directly resolvable by EIS, the overall HER mechanism in 1 M KOH likely involves parallel Volmer–Heyrovsky and Volmer–Tafel pathways, evident in the double phase-angle peak at low overpotential (−0.01 V *vs.* RHE). The clear resolution of both processes confirms efficient water dissociation (H_2_O → H* + OH^−^) on a-RuSi. Combined with insights from H* desorption and OH* adsorption kinetics, the disordered structure provides dual active sites that accelerate dissociation and decouple Volmer–Heyrovsky steps, surmounting typical constraints. This was further validated by *operando* Raman spectroscopy.

Potential-dependent *operando* Raman spectroscopy (Fig. 5d) monitored interfacial water restructuring during HER.^45,46^ Relative to the bulk electrolyte spectrum (Fig. 5e), the H–O–H bending mode (1643 cm^−1^ in bulk) red-shifted markedly to 1580 cm^−1^ under cathodic polarization (−0.4 V *vs.* RHE). This indicates strong a-RuSi–H_2_O interactions, weakening hydrogen bond networks. Consistent with the proposed Ru–Si dual-site mechanism—where Si^*δ*+^ serves as a Lewis acid for OH adsorption—the peak at 1064 cm^−1^ (attributed to symmetric Si–O stretching)^47,48^ intensified. Although no distinct Ru–H vibrations were observed, H_2_ rotational Raman bands (587 cm^−1^ and 815 cm^−1^) emerged at −0.2 V, confirming efficient H* recombination.^49^ No distinct Ru–O modes were observed, suggesting the absence of substantial Ru surface oxidation under these conditions. These findings are consistent with recent reports^50,51^ that disordered interfaces promote electrolyte interactions by inducing water ordering from random H_2_O clusters to aligned dipoles, thereby enhancing proton–electron transfer and lowering the water dissociation barrier.

### Theoretical insights into activity origins

Density functional theory (DFT) calculations were performed to elucidate the microscopic mechanism underlying the enhanced HER activity of a-RuSi, with emphasis on its dual active sites. First, electronic interactions between Ru and Si were examined by the PDOS calculations for a-RuSi, c-RuSi, and crystalline Ru (c-Ru) (Fig. 6a). Distinct peaks emerged between −6 and −10 eV in the Ru 4d orbitals of both a-RuSi and c-RuSi, but were absent in c-Ru. These features align with overlapping Ru 4d and Si 2s orbitals, as evidenced by matching signals in the Si 2s PDOS, indicating the presence of strong Ru–Si interactions.^33,52^ Bader charge analysis (Fig. 6b) revealed charge transfer from Si to Ru in c-RuSi and a-RuSi, consistent with XPS results (Fig. 2h and i). This resulted in negatively charged Ru and positively charged Si (Ru^*δ*−^–Si^*δ*+^) dual sites, respectively corresponding to the Lewis base and acid sites. Notably, a-RuSi's disordered structure induced unique charge redistribution, with Si donating less electron density to Ru compared to c-RuSi (Bader charge on Si: +0.45 |*e*| in a-RuSi *vs.* +0.81 |*e*| in c-RuSi). The distinct Ru^*δ*−^–Si^*δ*+^ pairs result in varying adsorption strengths for H* and OH*, influencing water dissociation barriers (Fig. 6c, d and S18–S20). Importantly, the presence of Si-to-Ru charge transfer alone does not determine the adsorption behavior of H* and OH*. In crystalline RuSi, the periodic Ru_*x*_Si_*y*_ structure imposes a relatively uniform and geometrically constrained local coordination environment. In contrast, the highly disordered a-RuSi structure provides a broader distribution of local Ru–Si coordination geometries and electronic environments, increasing the possibility of local configurations that more favorably balance H* and OH* binding. Thermal annealing progressively reorganizes these disordered local environments toward more ordered Ru_*x*_Si_*y*_ motifs, thereby changing their associated adsorption properties. These differences in local coordination and charge distribution are directly reflected in the calculated adsorption energetics and water-dissociation barriers. Specifically, c-Ru exhibits a high barrier (0.74 eV) and is characterized by strong H* adsorption (−0.56 eV) and weak OH* adsorption (−0.22 eV) (Fig. 6d and S18). The c-RuSi shows a similarly high barrier of 0.70 eV, but is characterized by an opposite adsorption profile, *i.e.* weak H* adsorption (−0.25 eV) and strong OH* adsorption (−0.55 eV) (Fig. 6d and S19). In contrast, a-RuSi achieves a substantially lower barrier (0.37 eV), enabled by more balanced adsorption energies for both H* (−0.50 eV) and OH* (−0.49 eV) (Fig. 6d and S20), thereby optimizing the overall energy landscape. This substantially reduced Volmer barrier suggests that water dissociation is no longer the kinetic bottleneck on a-RuSi, consistent with the experimentally observed shift of the rate-determining step toward the subsequent H* recombination/desorption process. It is noteworthy that the overall trend in adsorption energies aligns well with the corresponding experimental results (Fig. 4), confirming the decoupled binding behavior of H* and OH* in the Ru–Si system.

**Fig. 6:**
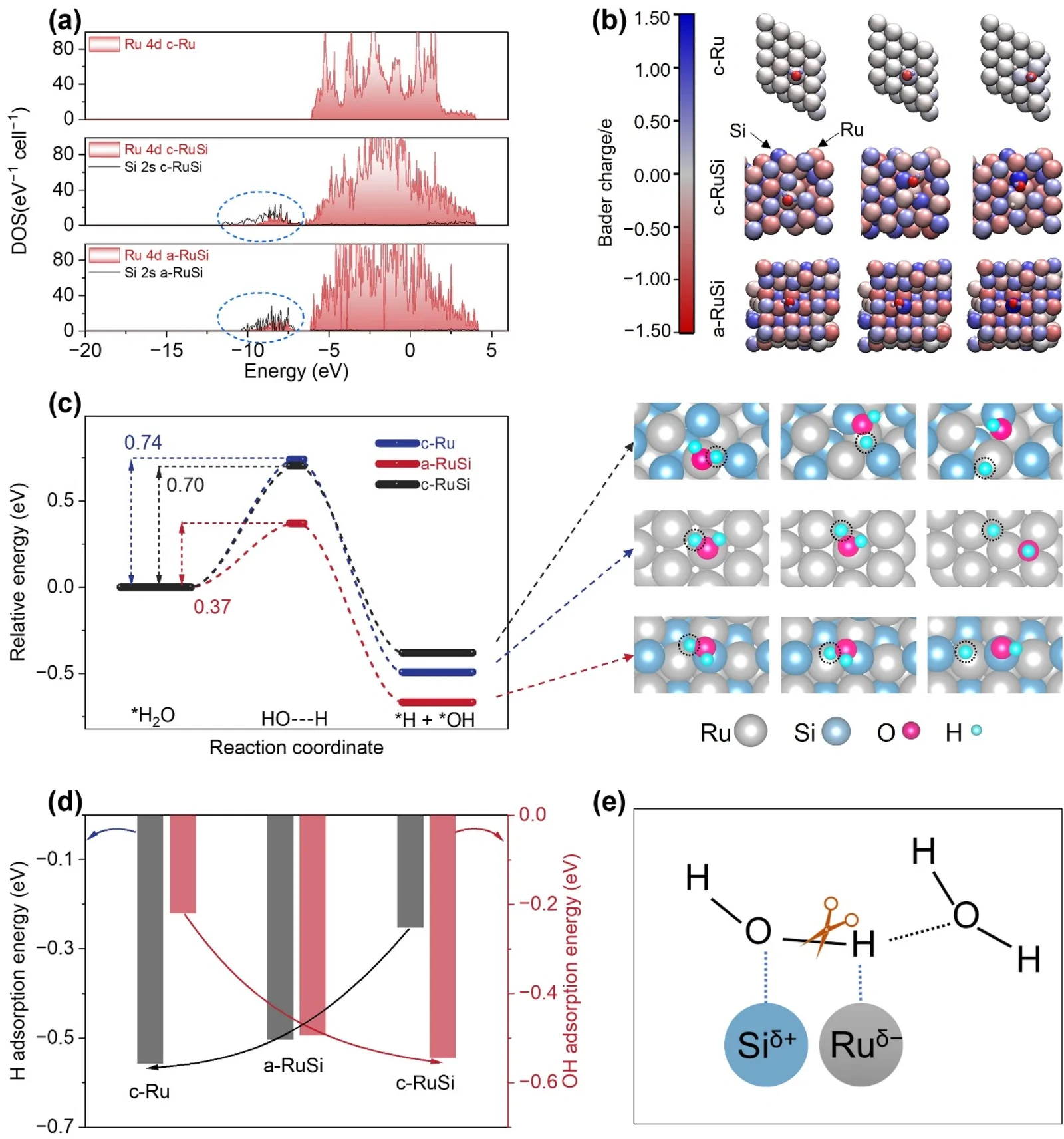
Theoretical insights into activity origins. (a) Projected density of states (PDOS) showing interactions between Si 2s and Ru 4d orbitals. (b) Bader charge analysis during water dissociation on c-Ru, c-RuSi, and a-RuSi. The red-blue scale bar indicates valence states from −1.50 to +1.50 e. (c) Energy profiles for water dissociation (left) and corresponding atomic configurations (right) depicting initial states (IS), transition states (TS), and final states (FS) for various catalysts. (d) Comparison of calculated adsorption free energies. (e) Schematic illustration of water dissociation at Ru–Si dual sites. The black line denotes the O–H bond, the blue dashed line represents adsorbate interactions with Ru–Si sites, and the black dashed line indicates a hydrogen bond.

To further clarify the influence of the local coordination environment on OH* adsorption, additional COHP analyses were performed for the optimized OH* adsorption configurations on c-Ru, a-RuSi, and c-RuSi (Fig. S21). On c-Ru, OH* adopts a threefold configuration involving three Ru atoms, with –ICOHP values of 1.40, 1.40, and 1.39 eV for O–Ru1, O–Ru2, and O–Ru3, respectively. In contrast, OH* adsorbs on top of a Si site on a-RuSi and forms a strong localized O–Si interaction with a –ICOHP value of 6.40 eV. On c-RuSi, OH* interacts simultaneously with Si and a neighboring Ru site, with –ICOHP values of 5.74 eV for O–Si and 0.93 eV for O–Ru. Thus, although the individual O–Si interaction is slightly stronger in a-RuSi, the additional O–Ru interaction in c-RuSi provides cooperative O–Si/O–Ru stabilization of OH*, consistent with its more negative OH* adsorption energy.

These results further show that, while the more positive Bader charge of Si in c-RuSi is consistent with its stronger Lewis-acid-like character and overall affinity toward OH-containing species, the actual OH* adsorption strength is also modulated by the local Ru–Si coordination environment and adsorption geometry. The crystalline Ru_*x*_Si_*y*_ motifs therefore promote stronger overall OH* stabilization, which can hinder hydroxyl removal and may reduce the availability of neighboring Ru sites for subsequent H*-related elementary steps. In contrast, a-RuSi provides a more balanced adsorption environment for H* and OH*, accounting for its superior overall HER kinetics.

Based on the preceding mechanistic insights, a schematic model illustrating water dissociation facilitated by the Ru–Si dual sites is presented (Fig. 6e). Water adsorption at these sites involves a concerted interaction in which the O^*δ*−^ atom interacts with the positively charged Si^*δ*+^ (Lewis acid), while the H^*δ*+^ atom interacts with the negatively charged Ru^*δ*−^ (Lewis base). This bifunctional adsorption, likely driven by both electrostatic and covalent interactions, effectively stabilizes the transition state. Collectively, this optimized bifunctional mechanism significantly lowers the water dissociation barrier, thereby enhancing the overall alkaline HER activity.

## Conclusions

In summary, we address the kinetic limitations of alkaline HER through a highly disordered Ru–Si dual-site catalyst platform. The chemical composition, geometric configurations, and electronic states of Ru–Si were systematically tuned by progressively tuning structural order through thermal annealing. Leveraging these dual sites, the Ru–Si catalyst series enables partially decoupled regulation of HBE and OHBE. The experimental descriptor analysis shows that the highest HER activity within this series is associated with a favorable balance between H* and OH* binding, supporting the ability of dual-site RuSi catalysts to access a higher-activity HBE–OHBE regime than conventional single-site catalysts. Accordingly, a-RuSi exhibits exceptional HER performance, with an overpotential of 7.85 mV at 10 mA cm^−2^ and stability for 1050 h at 1 A cm^−2^ in 1 M KOH, accompanied by enhanced water-dissociation and hydrogen-recombination kinetics. Atomic-scale mechanistic studies reveal that disorder engineering in a-RuSi generates diverse Ru^*δ*−^–Si^*δ*+^ Lewis acid–base pairs *via* ligand-to-metal charge transfer, promoting complementary adsorption of H* and OH* at Ru- and Si-containing sites. These results highlight the potential of disordered dual-site engineering for mitigating adsorption-energy trade-offs in multistep electrocatalytic reactions and provide a rational framework for the design of advanced bifunctional catalysts.

## Author contributions

J. W. proposed, designed and financed the project. J. W. and Y. L. co-supervised the research. W. C. conducted the materials synthesis and characterization. W. C. and C. L. performed the electrochemical experiments and analyzed the experimental data. H. G. and Y. L. performed the DFT calculations. W. C., H. G., Y. L. and J. W. co-wrote the paper. All authors discussed the results and commented on the manuscript.

## Conflicts of interest

The authors declare that they have no conflicts of interest.

## Supplementary Material

SC-OLF-D6SC05983J-s001

## Data Availability

All data generated or analyzed during this study are included in this published article and its supplementary information (SI) files. Supplementary information: experimental details, figures (including TEM, SEM, XRD, computational models, cyclic voltammetry measurements, CO stripping curves, and crystal orbital Hamilton population analyses), tables, and references supporting the main text. See DOI: https://doi.org/10.1039/d6sc05983j.
