# Supplementary material for: Amorphization-enabled decoupling of hydrogen and hydroxide binding on RuSi for efficient alkaline hydrogen evolution
Source: Chem Sci. 2026 Sep 28. Online ahead of print. doi: 10.1039/d6sc05983j (PMC13636003; doi:10.1039/d6sc05983j)
Supplement: Supplementary file 1 [file SC-OLF-D6SC05983J-s001.pdf]

Supplementary Information (SI) for Chemical Science.
This journal is © The Royal Society of Chemistry 2026
Supplementary Information (SI) for Chemical Science.
This journal is © The Royal Society of Chemistry

SUPPLEMENTARY INFORMATION

Amorphization-Enabled Decoupling of Hydrogen and Hydroxide Binding on RuSi for Efficient Alkaline Hydrogen Evolution

Weizheng Cai[1, 2], Haotian Guan[3, 4, 5 *], Chuanlong Liu[1], Yangfan Lu[3, 4 *], Jiazhen Wu[1, 6, 7 *]

[1] Department of Materials Science and Engineering, Southern University of Science and Technology, Shenzhen, 518055, China.

[2] School of Flexible Electronics, Sun Yat-sen University, Shenzhen, 518107, China.

[3] College of Materials Science and Engineering, National Engineering Research Center for Mg Alloys, National Key Laboratory of Advanced Casting Technologies, National Innovation Center for Industry-Education Integration of Energy Storage Technology, Chongqing University, Chongqing 400045, China.

[4] Chongqing Institute of New Energy Storage Materials and Equipment, Chongqing 401135, China.

[5] College of Materials Science and Engineering, Chongqing University of Technology, Chongqing 400054, China.

[6] Institute of Innovative Materials, Southern University of Science and Technology, Shenzhen 518055, China.

[7] State Key Laboratory of Quantum Functional Materials, Southern University of Science and Technology, Shenzhen, 518055, China.

*Corresponding author. E-mail: htguan@cqut.edu.cn, yangfanlu@cqu.edu.cn, wujz@sustech.edu.cn

Materials and methods

Chemicals

All chemicals were used as received without further purification. Potassium hydroxide (semiconductor grade, 99.99% trace metals basis) and Nafion^{TM} 117 solution (~5% in a mixture of lower aliphatic alcohols and water) were purchased from Sigma-Aldrich. Lanthanum (99.9%) and silicon (99.999%) were obtained from Thermo Scientific, while ruthenium (99.95%) was sourced from PrMat Co., Ltd.

Materials preparation

LaRuSi was synthesized by arc-melting stoichiometric amounts of lanthanum, ruthenium, and silicon ingots under an argon atmosphere. The melting process was repeated five times to ensure homogeneity, with weight loss below 0.1%. The resulting silver-colored ingots were ground into powder using an agate mortar in an Ar-filled glovebox. As the as-melted LaRuSi was not single-phase, it was annealed at 1000 °C for 10 days to eliminate impurity phases.

LaRuSi was chemically etched to remove lanthanum by immersing ~0.2 g of powder in 10 mL of 1 M HCl in a centrifuge tube. The mixture was stirred for 24 h, centrifuged to separate the solid, washed three times each with distilled water and ethanol, and vacuum-dried at room temperature for 12 h. The a-RuSi sample was then prepared from this acid etched product using a similar procedure but with 6 M KOH as the etchant for 42 h. The structural order of a-RuSi was tuned by annealing under an

argon atmosphere at temperatures ranging from 200 to 700 °C for 60 min, with progressive ordering occurring as the annealing temperature increased.

Materials characterization

Powder X-ray diffraction (XRD) patterns were collected on a Bruker D2 Phaser diffractometer using Cu Kα radiation (λ = 1.5418 Å). Sample morphology was examined by field emission scanning electron microscopy (FE-SEM, TESCAN MIRA3). Specific surface areas were measured via Brunauer–Emmett–Teller (BET) analysis of N_2 adsorption–desorption isotherms at −196 °C on a Micromeritics ASAP 2460 analyzer. High-angle annular dark-field scanning transmission electron microscopy (HAADF-STEM) and bright-field scanning transmission electron microscopy (BF-STEM) images were captured using a JEOL JEM-ARM300F atomic resolution analytical electron microscope operated at 200 kV. Elemental compositions were determined by energy dispersive X-ray spectroscopy (EDS). Surface chemical analyses were conducted by X-ray photoelectron spectroscopy (XPS) on a Thermo Scientific ESCALAB Xi+ spectrometer with monochromatic Al Kα radiation (1486.6 eV); binding energies were calibrated to the C 1s peak at 284.8 eV.

Electrochemical measurements

All electrochemical experiments were performed at room temperature using a CHI 760E workstation (Shanghai CH Instruments Co., China) in a standard three-electrode cell. The working electrode was a 5 mm diameter glassy carbon rotating disk electrode (PINE). A carbon rod and Ag/AgCl electrode (PINE, saturated with KCl and calibrated

against a standard hydrogen electrode) served as the counter and reference electrode, respectively. The potential was calculated using the following equation:

$$E(\text{RHE}) = E(\text{Ag/AgCl}) + 0.196 + 0.059 \times \text{p} \quad (1)$$

Working electrodes were prepared by dispersing 5 mg of catalyst in 480 μL deionized water, 480 μL isopropanol, and 40 μL Nafion™ 117 solution. The mixture was sonicated for 1 h, and 10 μL of the catalyst ink was drop-cast onto a precleaned glassy carbon disk (loading: 250 μg cm^{-2}). The electrode was air-dried at room temperature for more than 1 h.

Prior to data collection, working electrodes were cycled continuously between −0.04 and 0.2 V vs RHE until reproducible voltammograms were obtained (room temperature, 1600 rpm, 100 mV s^{-1} scan rate). Solution resistance (Rs) was determined from Nyquist plots and was applied for iR compensation using $E_c = E_m - iR_s$, with E_c as the corrected potential and E_m as the measured potential. Hydrogen evolution reaction activity was evaluated via linear sweep voltammetry at 10 mV s^{-1} in 1 M KOH. Electrochemical impedance spectroscopy was conducted using the same CHI 760E workstation. Spectra were acquired at various DC potentials over a frequency range from 0.1 Hz to 100 kHz, with a 10 mV sinusoidal potential amplitude.

High-current stability test

The long-term chronopotentiometric stability test was conducted in a conventional H-cell containing 80 mL of 1 M KOH electrolyte in each compartment. The catalyst was

loaded on carbon paper with a geometric area of 0.5 × 0.5 cm^2. The applied current density was 1000 mA cm^{-2}, corresponding to a total current of 250 mA. The electrolyte was not operated in a flow configuration. The test was performed under ambient laboratory conditions near room temperature, without external heating or active temperature control. During long-term operation, deionized water was replenished approximately every five days to compensate for water consumption and evaporation and to maintain the electrolyte level. The KOH electrolyte was neither replaced nor replenished during the test; only deionized water was periodically added. Under these static high-current conditions, transient local pH and K^+ concentration gradients and gas-bubble accumulation/release cannot be completely excluded and may contribute to short-term potential fluctuations. The complete raw potential–time curve is provided in **Fig. S8a**.

Operando Raman spectroscopy

Operando Raman measurements were conducted using a LabRAM HR Evolution spectrometer (Horiba) coupled to an electrochemical workstation. The custom Teflon electrochemical cell featured a quartz window between the sample and the Raman objective lens. The working electrode was inserted through the cell wall, with its surface oriented perpendicular to the laser beam. A platinum wire and Ag/AgCl electrode served as the counter and reference electrodes, respectively. Spectra were acquired in 1 M KOH at applied potentials ranging from 0.08 to −0.4 V vs RHE. Each spectrum was recorded after 60 s at the target potential, with an acquisition time of 42 s to prevent

laser-induced catalyst damage. To minimize signal drift, spectra were collected across three Raman shift windows (500–1000 cm^{-1}, 1000–1250 cm^{-1}, and 1450–1800 cm^{-1}) using an 1800 grooves mm^{-1} grating.

Computational details

First-principles DFT calculations were conducted using the projector-augmented wave (PAW) method as implemented in the Vienna Ab initio Simulation Package (VASP).[1, 2] The Perdew-Burke-Ernzerhof (PBE) functional within the generalized gradient approximation (GGA) was employed[1, 3, 4]. Van der Waals interactions were incorporated via the DFT-D3 correction scheme in conjunction with PBE-PAW potentials. A Γ-centered 5 × 5 × 1 k-point mesh was used for all simulations, with a plane-wave cutoff energy of 520 eV. Atomic positions were relaxed until forces were below 0.01 eV $Å^{-1}$. A 20 Å vacuum layer was included to prevent periodic interactions between slabs. Pure Ru models were constructed on the (001) surface using a 6 × 6 supercell with four atomic layers. For Ru–Si systems, c-RuSi models were built on the (020) surface of Ru_4Si_3 using a 1 × 2 supercell, in accordance with our previous finding that Ru_4Si_3 forms in high-temperature annealed samples[5]. Furthermore, XPS analysis of the annealed samples indicated a decrease in the surface Ru concentration, leading to partial removal of surface Ru atoms in the model. The a-RuSi models were derived by selectively removing Si atoms from the bulk of the La-eliminated LaRuSi structure, with the surface Ru:Si ratio fixed at 1:1. This ratio was chosen to construct an idealized Si-containing local environment in which adjacent Ru and Si sites can be explicitly

examined, rather than to reproduce the average composition of the experimental catalyst. After full structural relaxation of the 3 × 3 models, the final a-RuSi configuration was obtained (**Fig. S22**). This model is therefore used to represent a disordered local Ru–Si motif rather than a compositionally faithful or unique atomistic reconstruction of the experimental a-RuSi surface.

Adsorption energies (E_{ads}) were calculated as

$$E_{ads} = E_{total} - E_{sub} - E_{adsorbate} \quad (2)$$

where E_{total} is the total energy of the adsorbate-covered slab, E_{sub} is the energy of the bare slab, and $E_{adsorbate}$ is the energy of the isolated adsorbate. For hydrogen, $E_H = 1/2\ E_{H2}$ (gas-phase H_2); For hydroxide, $E_{OH} = E_{H2O} - 1/2E_{H2}$. Transition states and minimum energy paths for H_2O dissociation were determined using the climbing-image nudged elastic band (CI-NEB) method. Density of states (DOS) was calculated for a-RuSi, c-RuSi and c-Ru to investigate their electronic structures. Bader charge analysis[6, 7] was used to evaluate electronic charge densities, with visualizations of charge redistribution before and after H_2O dissociation.

Supporting figures and tables

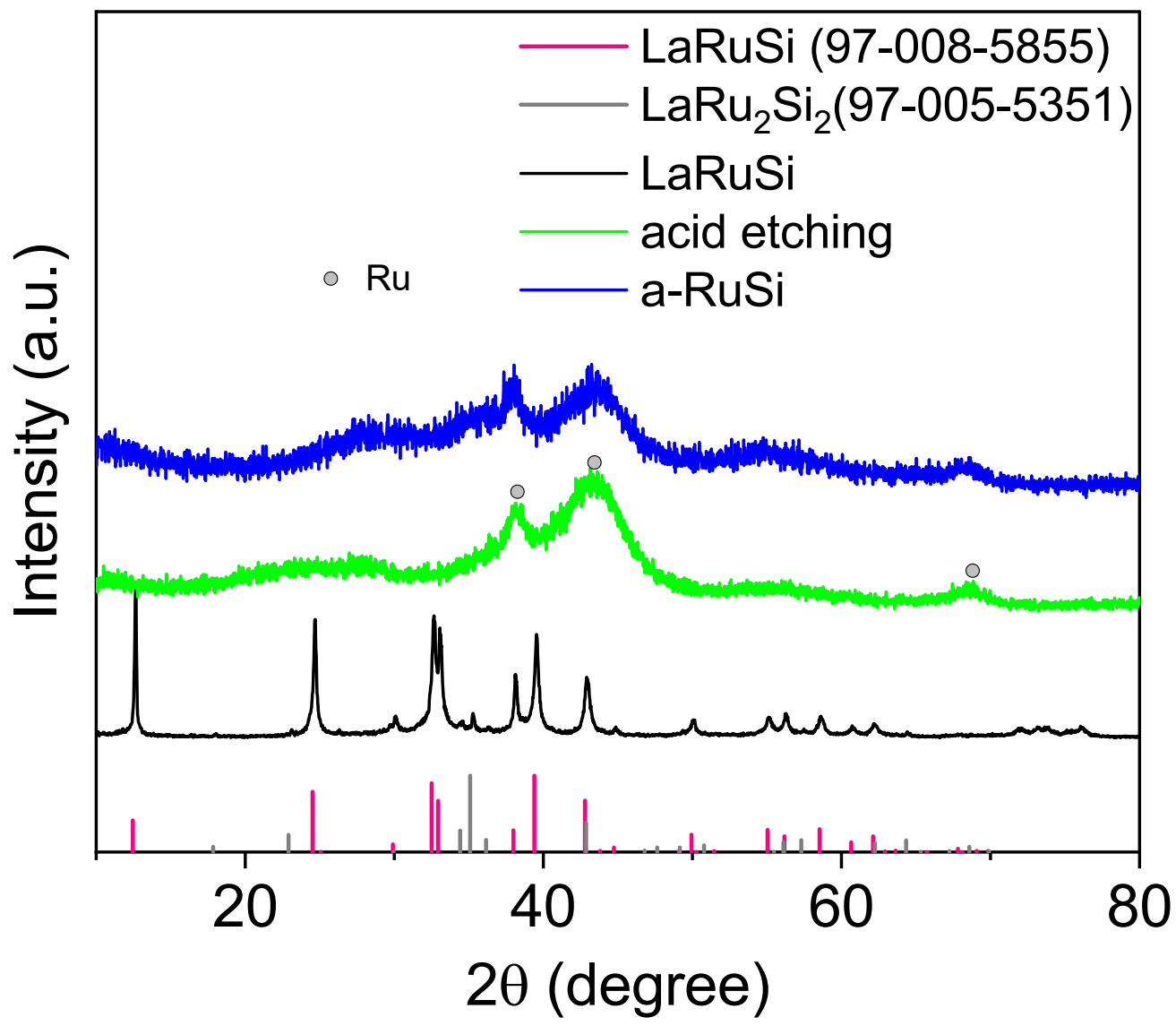

Fig. S1. X-ray diffraction (XRD) patterns of crystalline LaRuSi precursor, acid-etched LaRuSi, and amorphous RuSi (a-RuSi) formed on particle surfaces after alkaline etching of the acid-etched LaRuSi.

Note: Acid etching selectively removes La, while subsequent alkaline etching removes SiO_x, yielding a-RuSi and Ru metal as the primary residues.

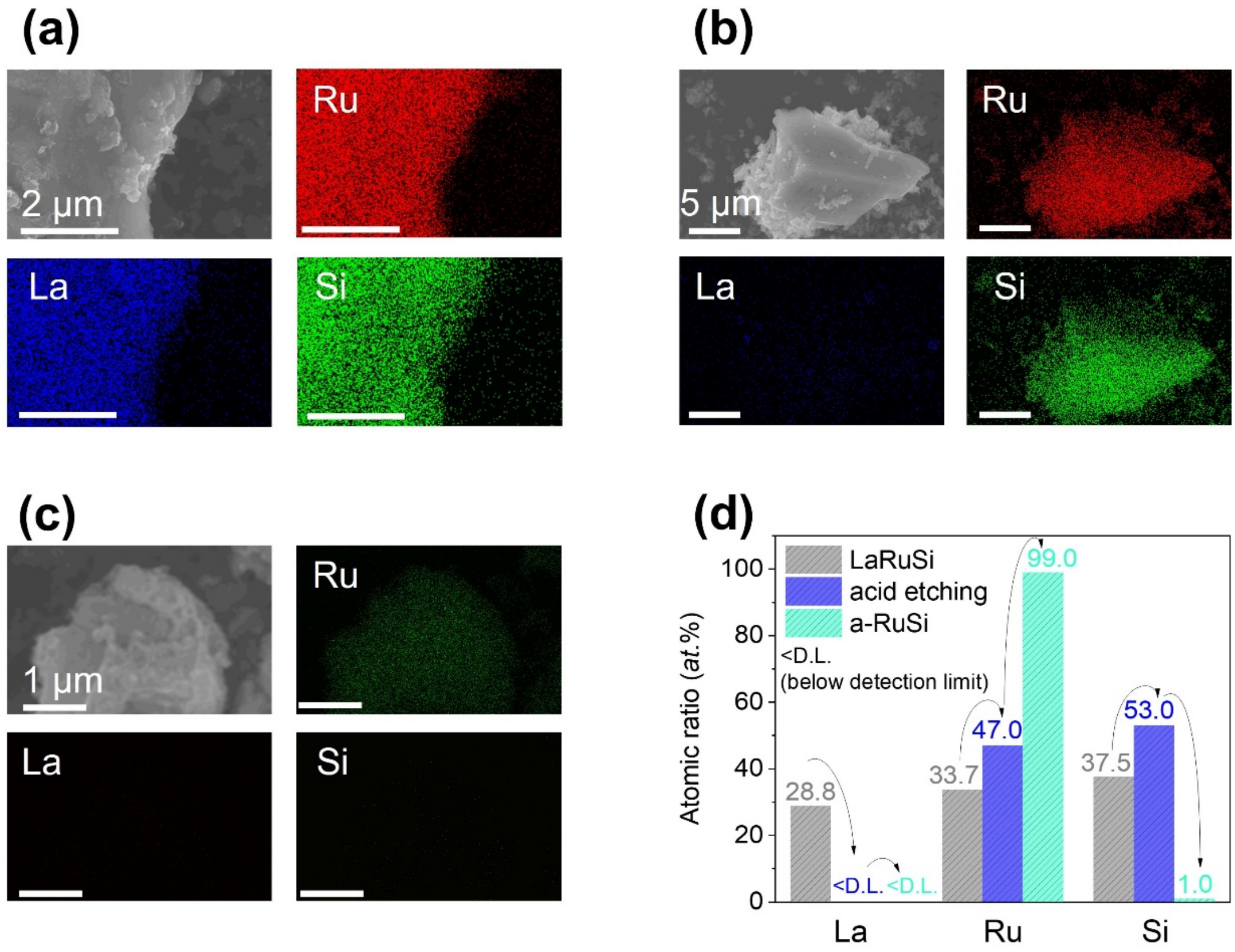

Fig. S2. Scanning electron microscopy (SEM) images with corresponding energy-dispersive X-ray spectroscopy (EDS) mappings of (**a**) crystalline LaRuSi precursor, (**b**) acid-etched LaRuSi, and (**c**) a-RuSi. **d**, Elemental composition quantified by EDS analysis.

Note: Acid etching completely removed La, resulting in a composition of 47 *at.*% Ru and 53 *at.*% Si. Subsequent alkaline etching further modified the composition, increasing Ru to 99 *at.*% and reducing Si to 1 *at.*%. This significant Si depletion indicates that alkaline etching selectively removes unstable silicon species, forming a distinctive a-RuSi structure.

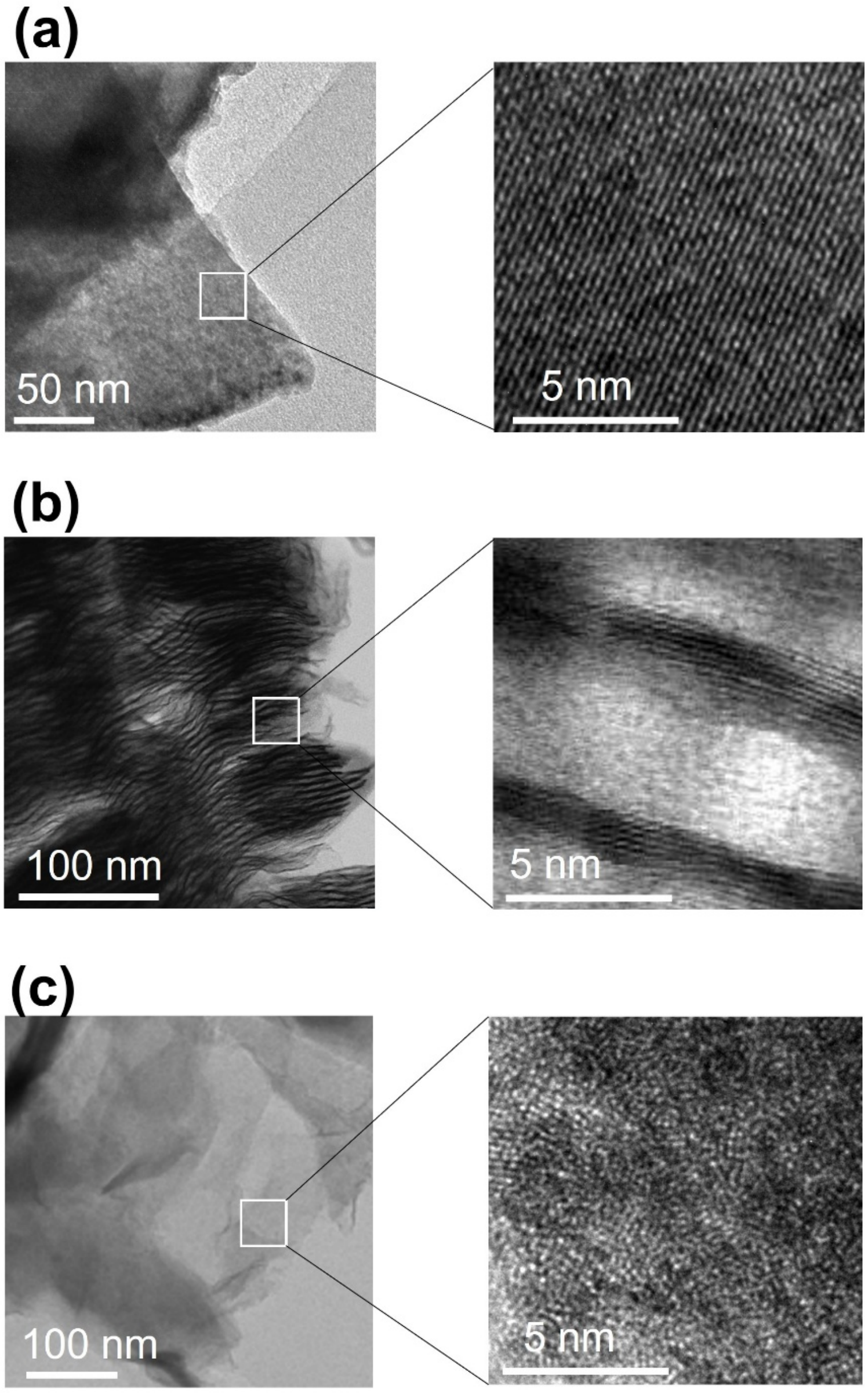

Fig. S3. High-resolution transmission electron microscopy (HRTEM) images of (**a**) LaRuSi precursor, (**b**) acid-etched LaRuSi, and (**c**) a-RuSi.

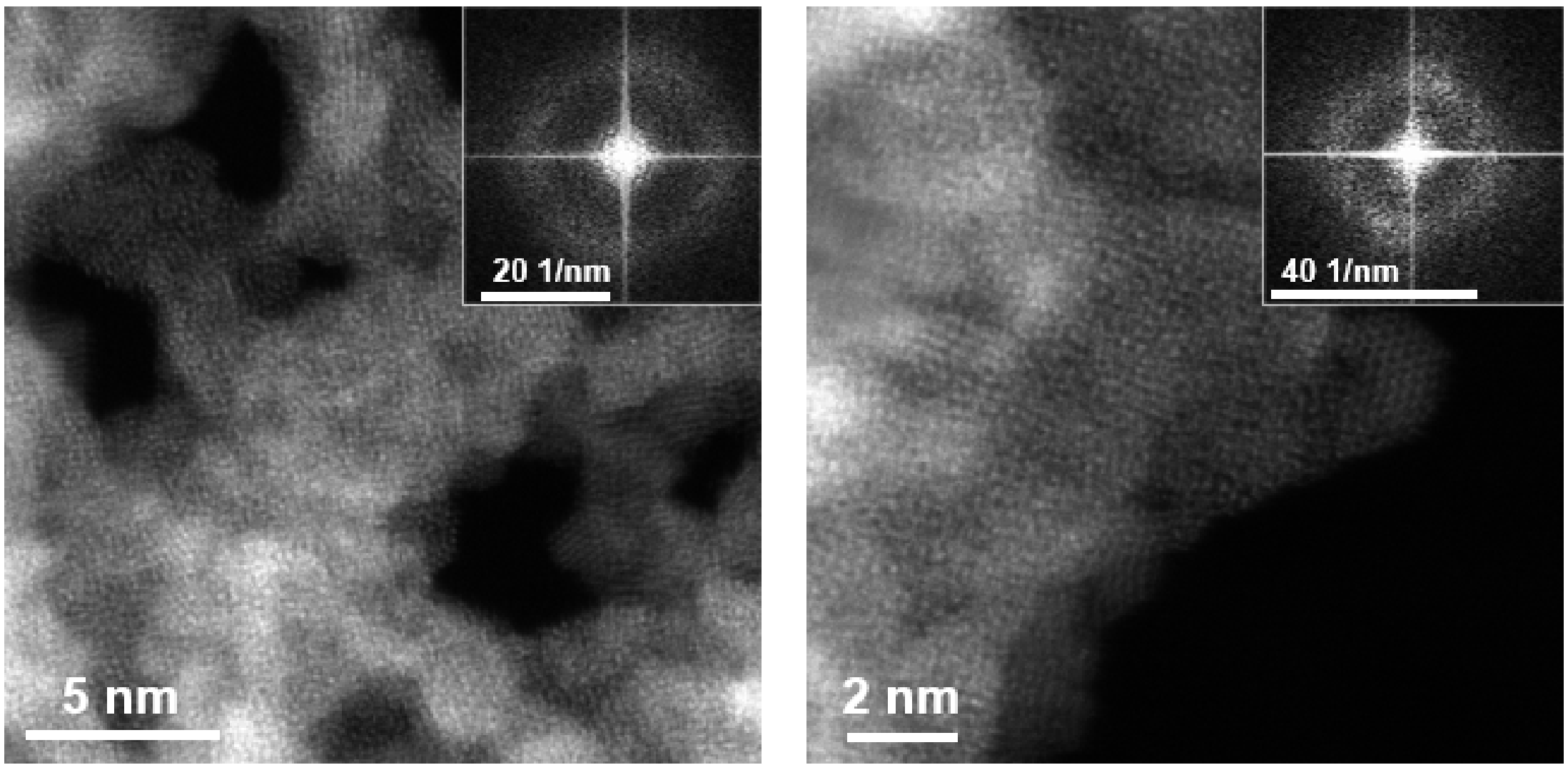

Fig. S4. Additional aberration-corrected HAADF-STEM images of pristine a-RuSi collected from different regions. The images show predominantly nonperiodic atomic contrast without extended lattice fringes or well-defined crystalline domains, supporting the highly disordered/amorphous nature of pristine a-RuSi in the examined regions.

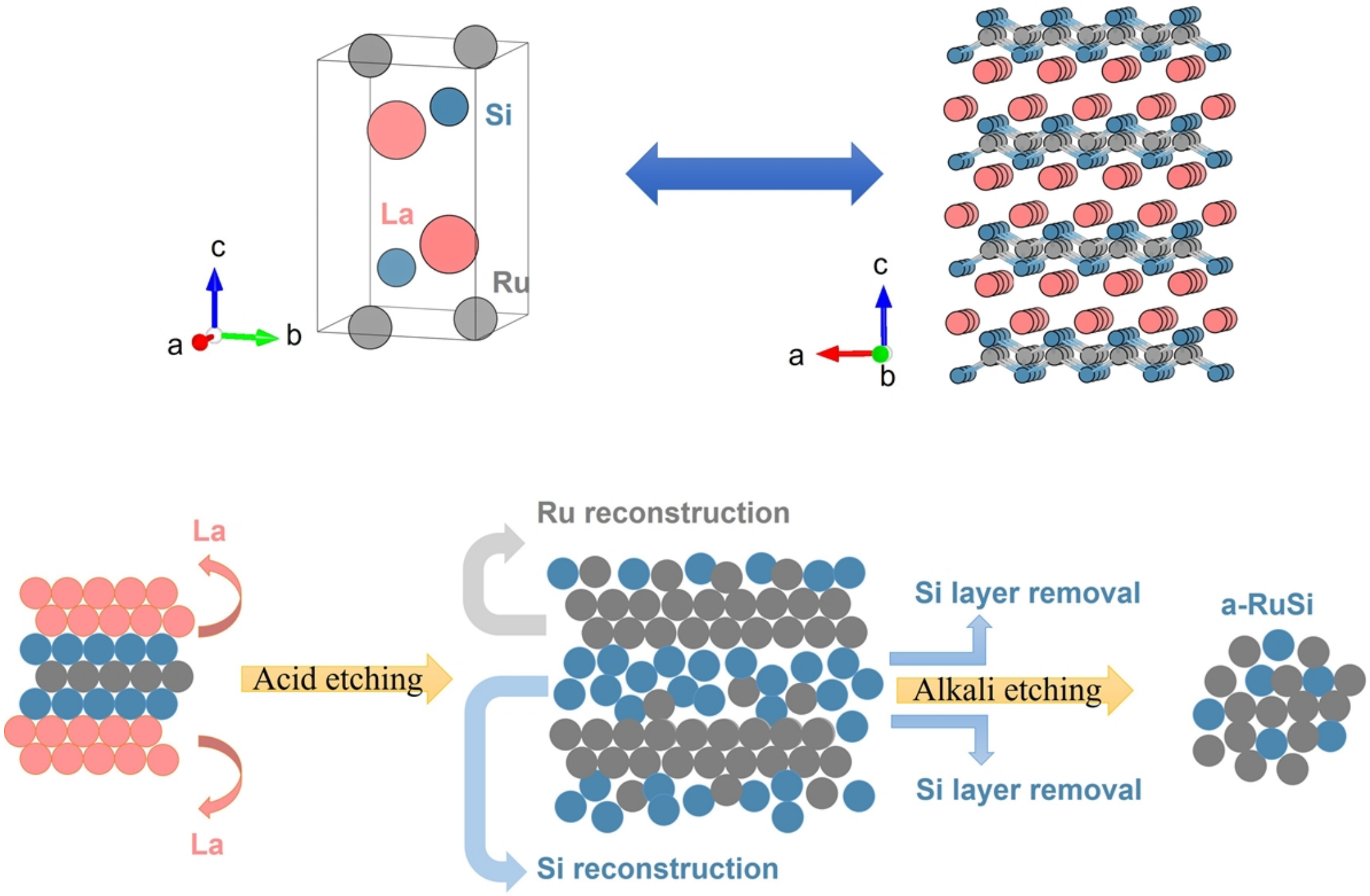

Fig. S5. Schematic illustration of the two-step selective etching process at atomical scale: acid leaching of La from crystalline LaRuSi precursor to form reconstructed Ru/Si heterostructure, followed by alkaline etching of Si to achieve a-RuSi.

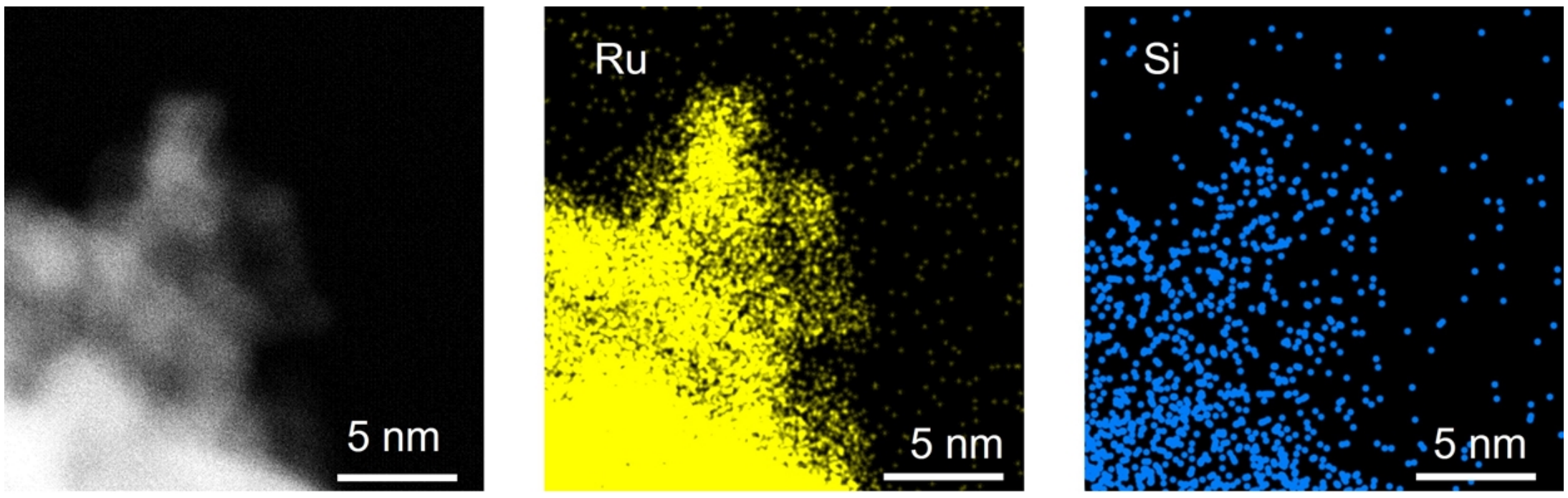

Fig. S6. Aberration-corrected TEM (AC-TEM) image and corresponding elemental mappings of a-RuSi.

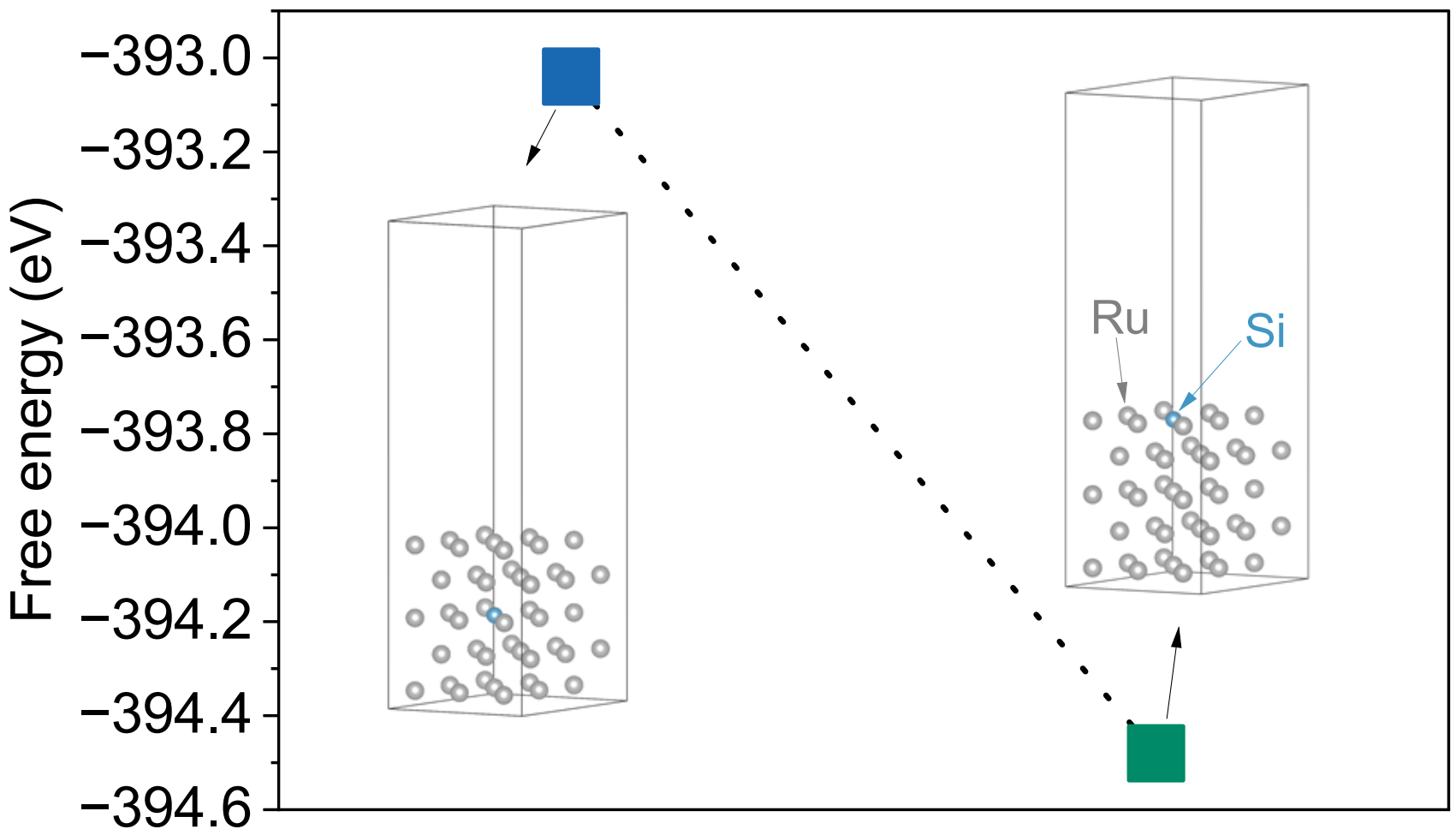

Fig. S7. Stability comparison of crystalline Ru system with internally distributed versus surface-localized silicon. This calculation illustrates Si surface segregation in the Ru-Si system during high-temperature annealing.

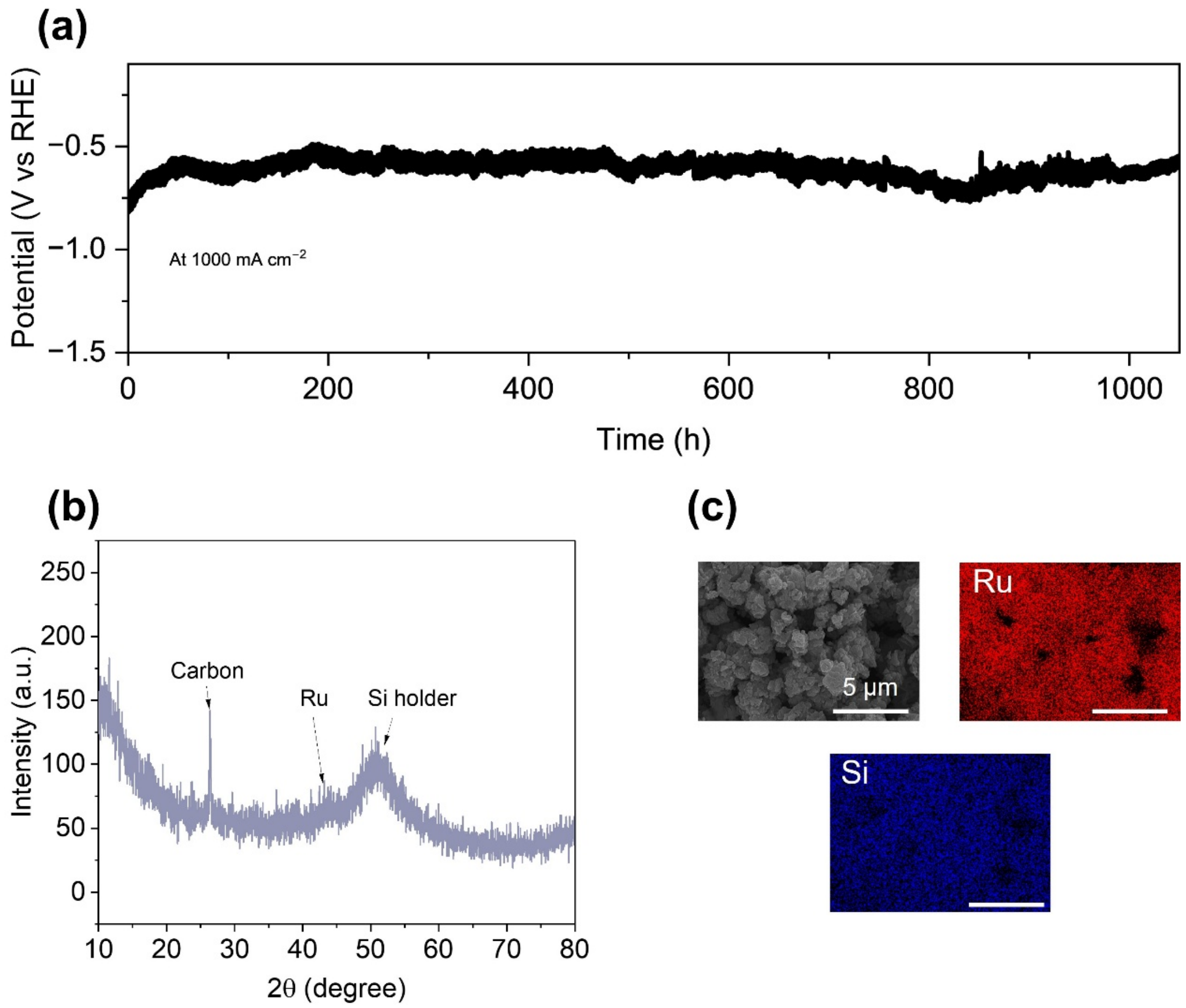

Fig. S8. (**a**) Complete raw chronopotentiometric curve of a-RuSi at 1000 mA cm^{-2} in 1 M KOH for 1050 h using an H-cell configuration. During the test, deionized water was replenished approximately every five days to compensate for water consumption and evaporation and to maintain the electrolyte level. The small potential fluctuations are likely associated with gas-bubble accumulation/release and manual replenishment events, although individual fluctuations cannot be unambiguously assigned because the test was not continuously visually monitored. (**b**) XRD patterns and (**c**) SEM-EDS elemental mappings of a-RuSi after 1050-hour stability testing at 1000 mA cm^{-2} in 1 M KOH. The retained highly disordered structure and homogeneous elemental distribution demonstrate substantial structural stability after prolonged high-current operation.

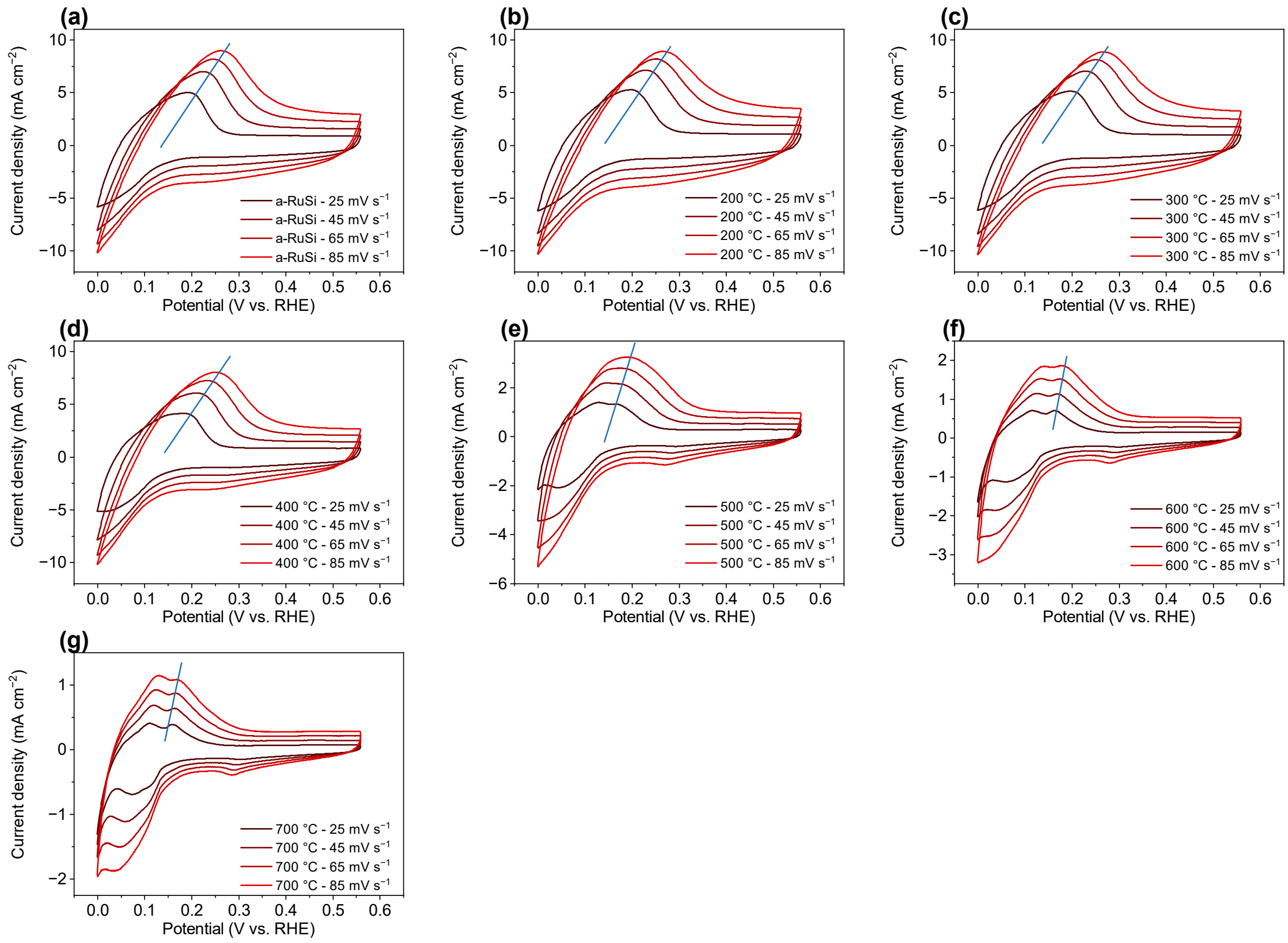

Fig. S9. Hydrogen underpotential deposition (H-UPD) cyclic voltammetry at varied scan rates (25–85 mV s^{-1}) in Ar-saturated 0.1 M KOH. **a**, a-RuSi, unannealed. **b**, a-RuSi annealed at 200 °C. **c**, a-RuSi annealed at 300 °C. **d**, a-RuSi annealed at 400 °C. **e**, a-RuSi annealed at 500 °C. **f**, a-RuSi annealed at 600 °C. **g**, a-RuSi annealed at 700 °C.

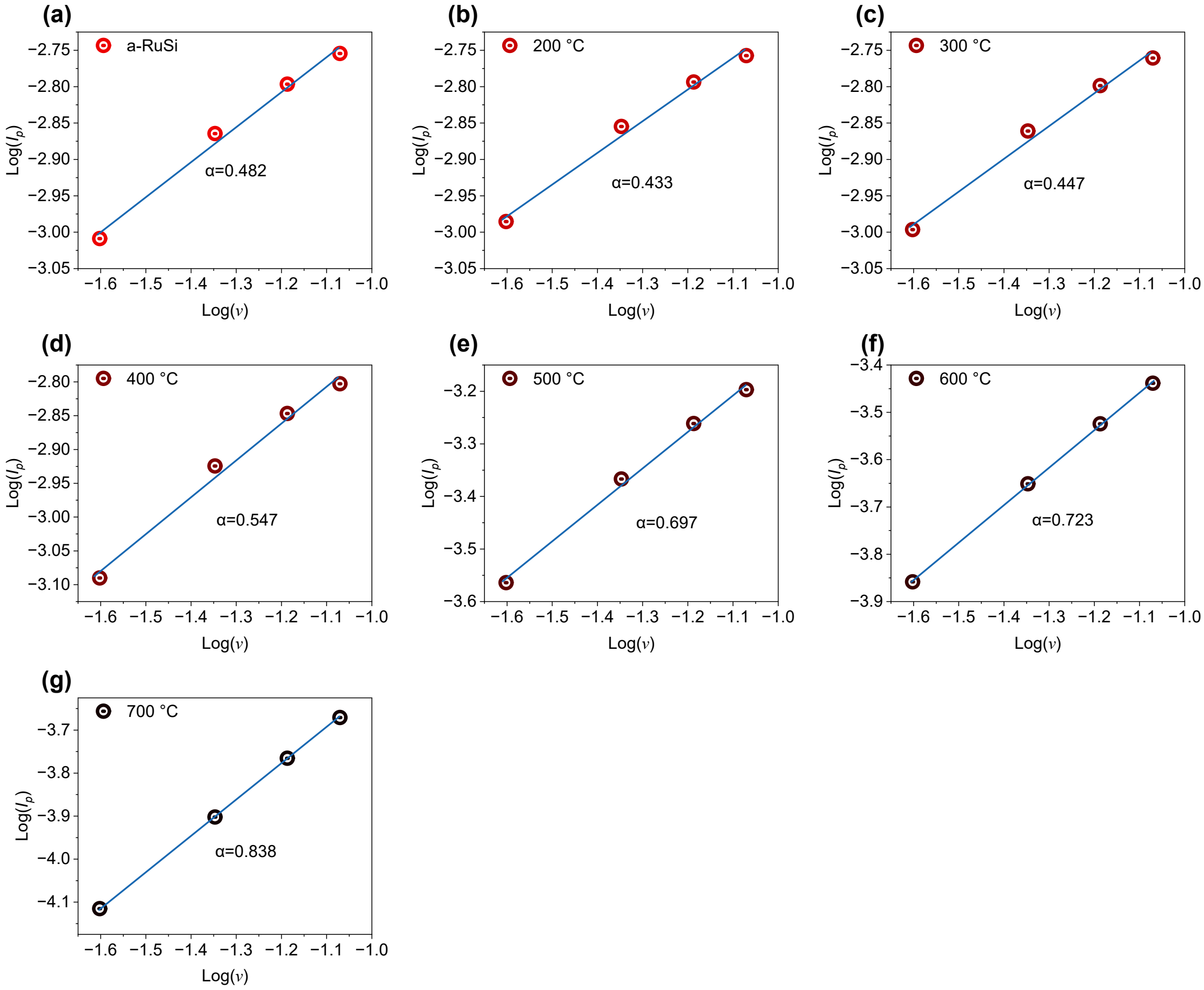

Fig. S10. Plots of log(I_p) versus log(v) derived from H-UPD cyclic voltammetry in **Fig. S12**. **a**, a-RuSi, unannealed. **b**, a-RuSi annealed at 200 °C. **c**, a-RuSi annealed at 300 °C. **d**, a-RuSi annealed at 400 °C. **e**, a-RuSi annealed at 500 °C. **f**, a-RuSi annealed at 600 °C. **g**, a-RuSi annealed at 700 °C.

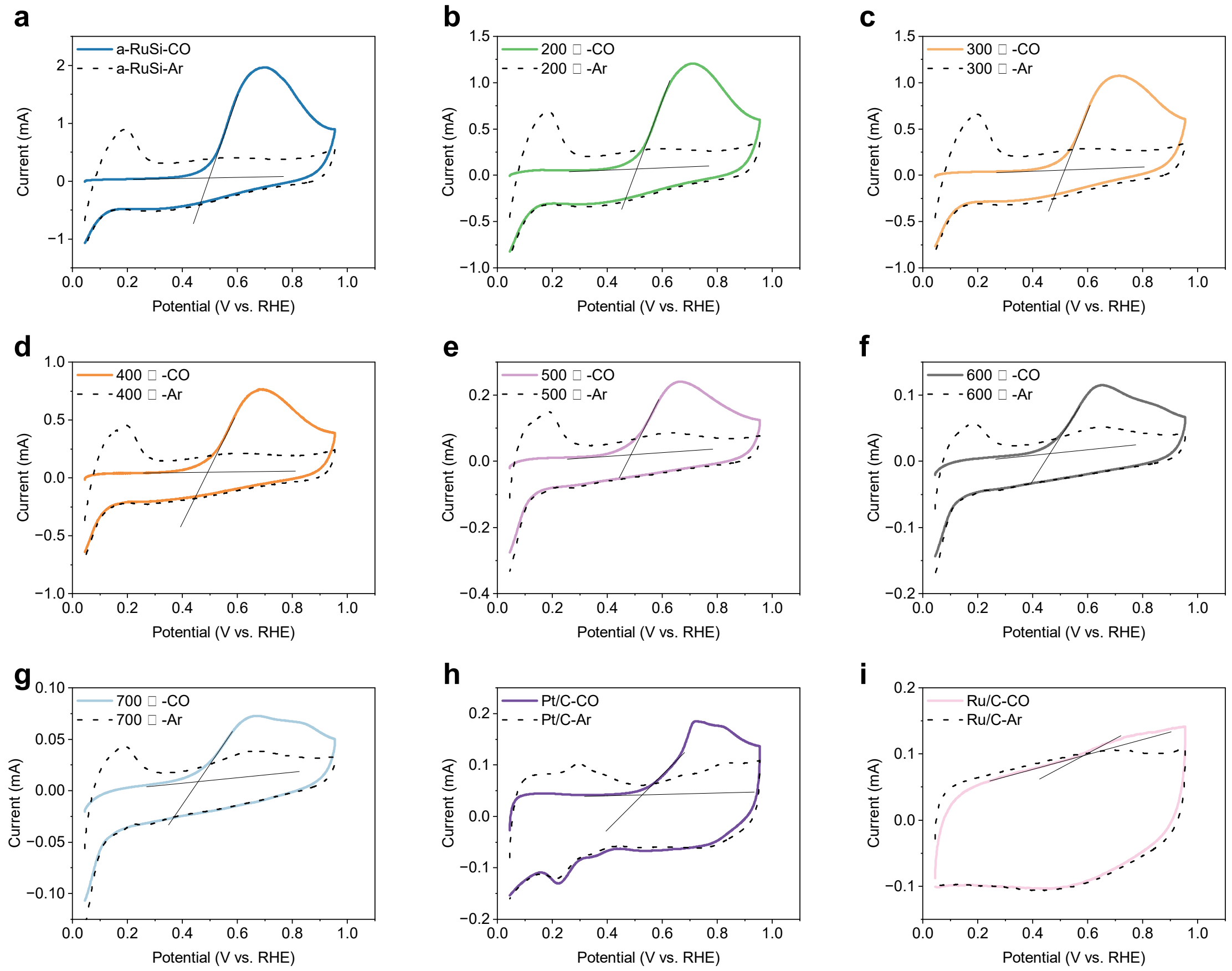

Fig. S11. CO stripping curves for (a) a-RuSi, (b) 200 °C, (c) 300 °C, (d) 400 °C, (e) 500 °C, (f) 600 °C, (g) 700 °C, (h) commercial Pt/C, and (i) commercial Ru/C.

Note: The solid lines represent the CO-covered surface profiles, while the dashed lines correspond to the Ar-saturated baseline measurements. The CO oxidation/desorption onset potential was determined from the intersection of tangents drawn to the baseline and the rising oxidation feature. Prior to CO adsorption, the electrode was stabilized by two cyclic voltammetry scans between 0.1 and 1.0 V vs. RHE in 0.1 M KOH. CO was then introduced by bubbling at a fixed potential of 0.1 V vs. RHE for 10 min to allow adsorption on the catalyst surface. The electrolyte was subsequently purged with high-purity Ar for 25 min at the same potential to remove dissolved and physically adsorbed CO while retaining chemisorbed CO on the electrode surface. Finally, two potential sweeps between 0.1 and 1.0 V vs. RHE were recorded to obtain the characteristic CO oxidation/desorption features.

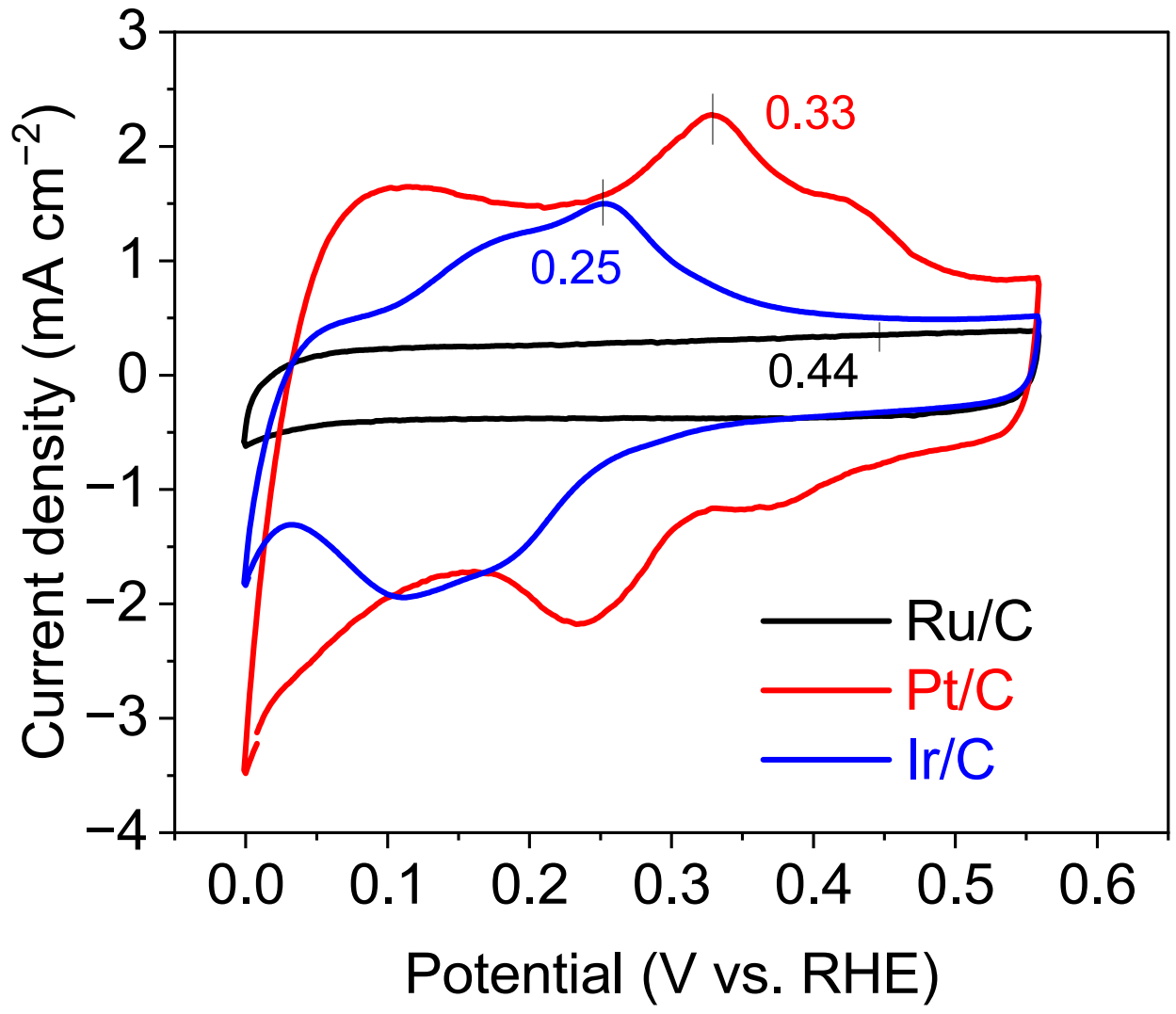

Fig. S12. H-UPD cyclic voltammetry profiles at a scan rate of 25 mV s^{-1} in Ar-saturated 0.1 M KOH for various commercial catalysts (Ru/C, Pt/C, and Ir/C). All the commercial catalysts show relatively higher H_{des} than our prepared catalysts.

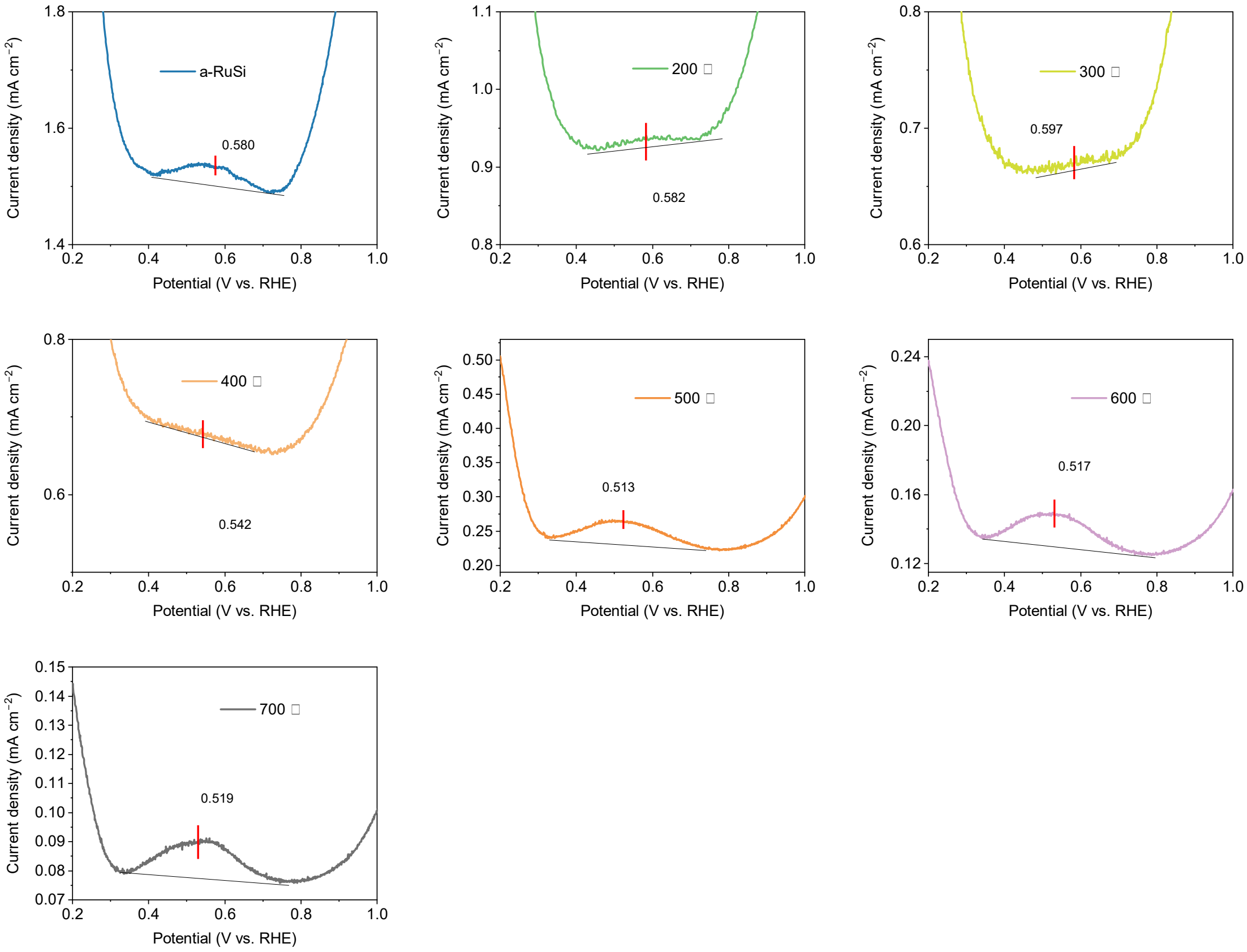

Fig. S13. Determination of E_{OHads} from hydroxide adsorption curves for a-RuSi and annealed RuSi catalysts. For all samples, the local baseline was estimated from the adjacent double-layer regions and subtracted using the same procedure. The red vertical markers indicate the assigned E_{OHads} values, determined from the maxima of the baseline-corrected OH adsorption features. The extracted E_{OHads} values are used as semi-quantitative descriptors of the relative OH* adsorption tendency within the RuSi catalyst series.

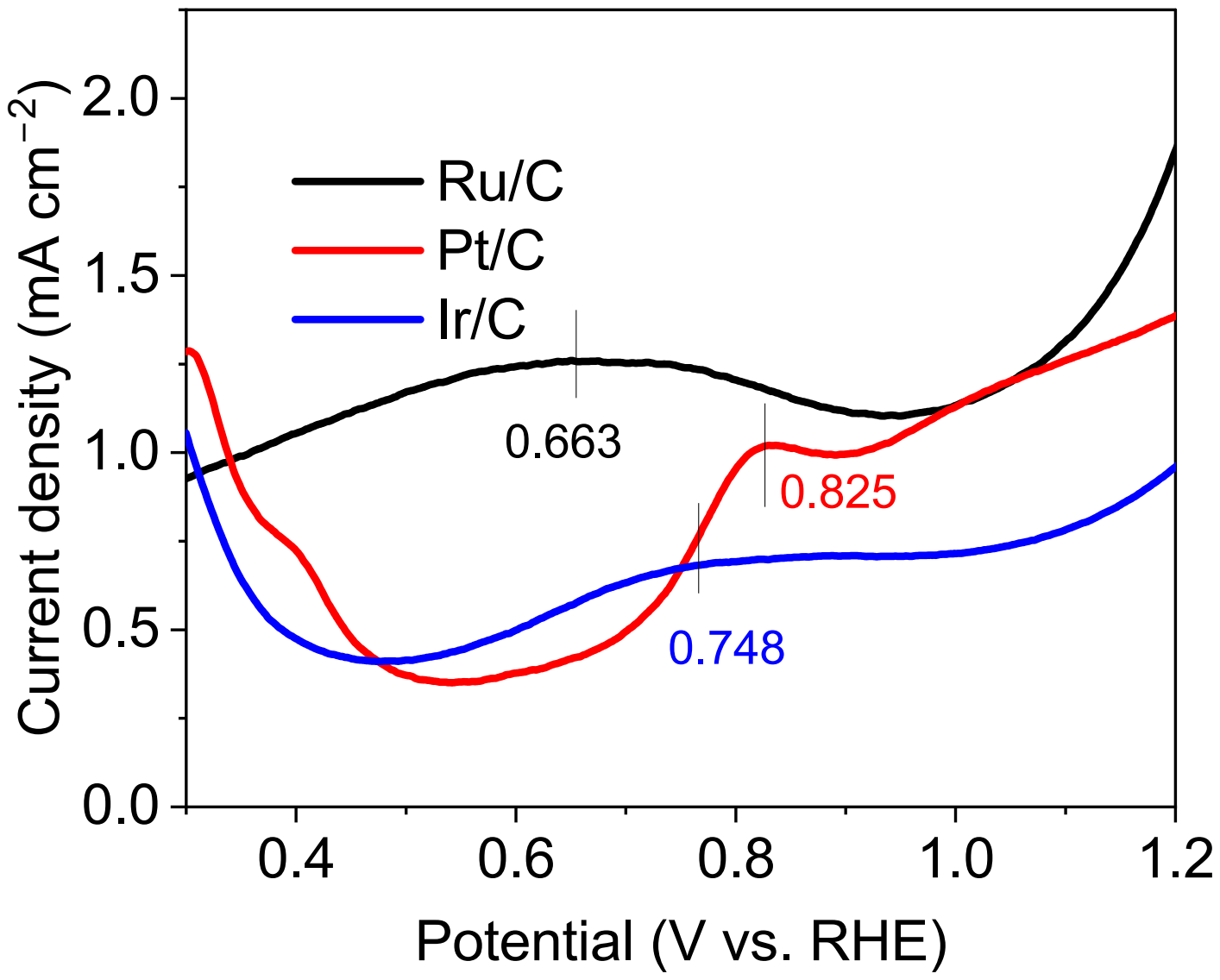

Fig. S14. OH adsorption curves at a scan rate of 25 mV s^{-1} in Ar-saturated 0.1 M KOH for commercial catalysts (Ru/C, Pt/C, and Ir/C).

Note: Our results are consistent with prior experimental observations [8] and computational predictions [9].

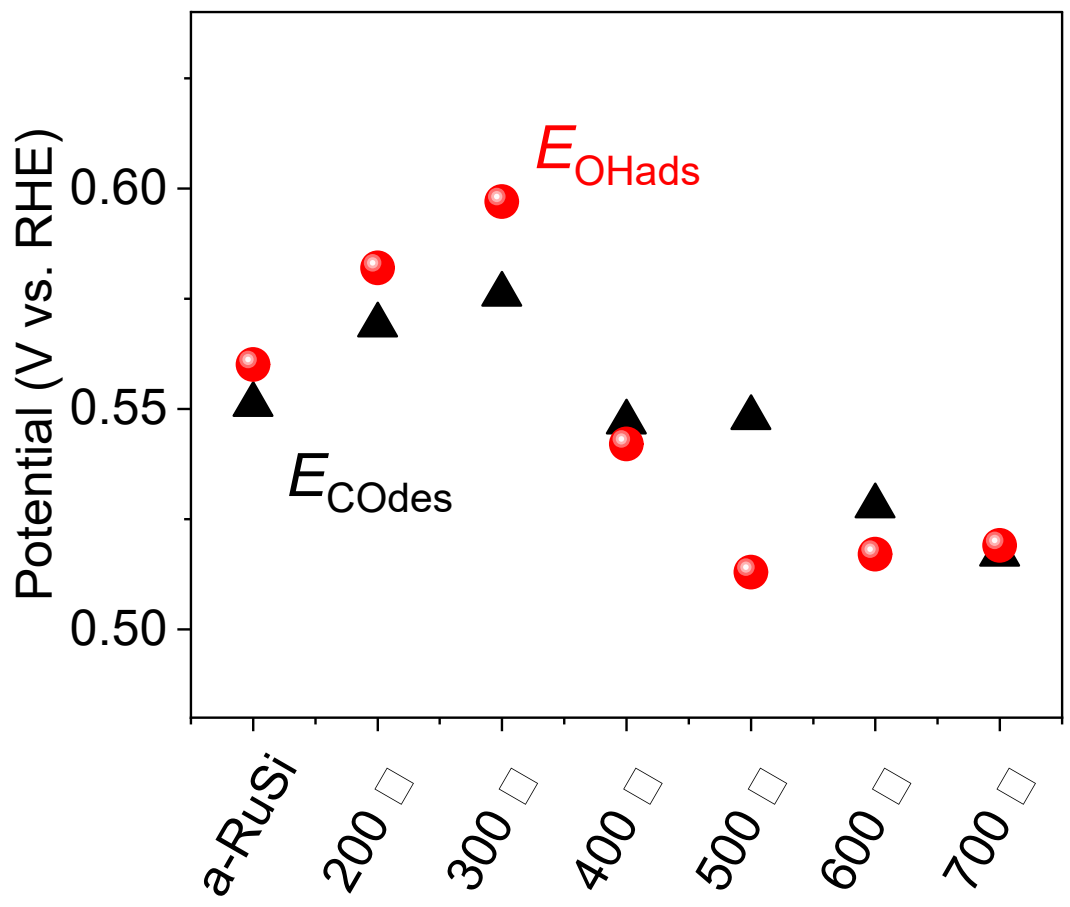

Fig. S15. Comparison of E_{COdes}, determined from the CO oxidation/desorption onset potentials in **Fig. S11**, and E_{OHads}, extracted from the hydroxide adsorption peak potentials in **Fig. 4d**, for the RuSi catalyst series. The similar overall trends of E_{COdes} and E_{OHads} provide independent support for using E_{OHads} as a relative experimental descriptor of OH* adsorption within the RuSi series.

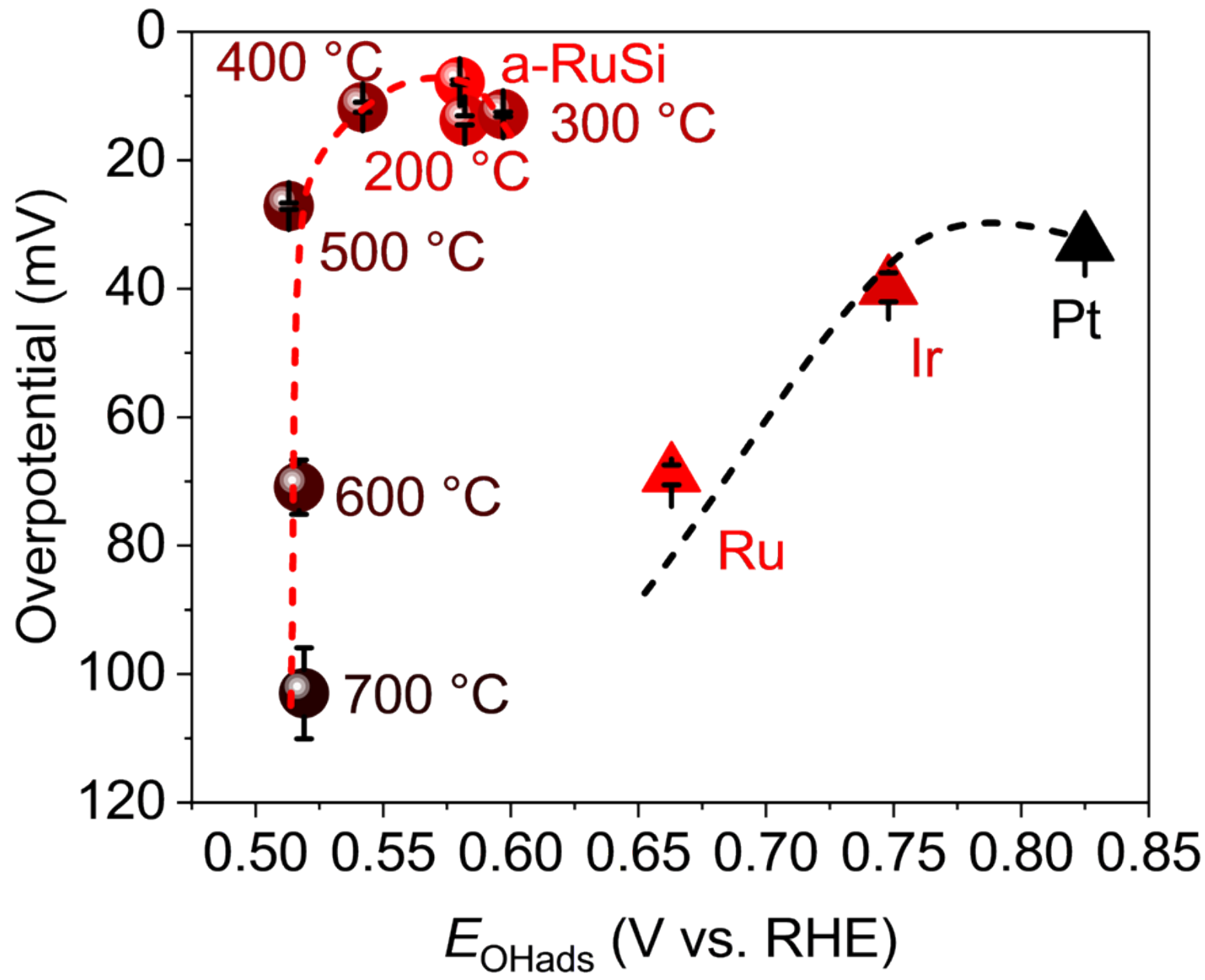

Fig. S16. Activity volcano plot correlating E_{OHads} with HER activity.

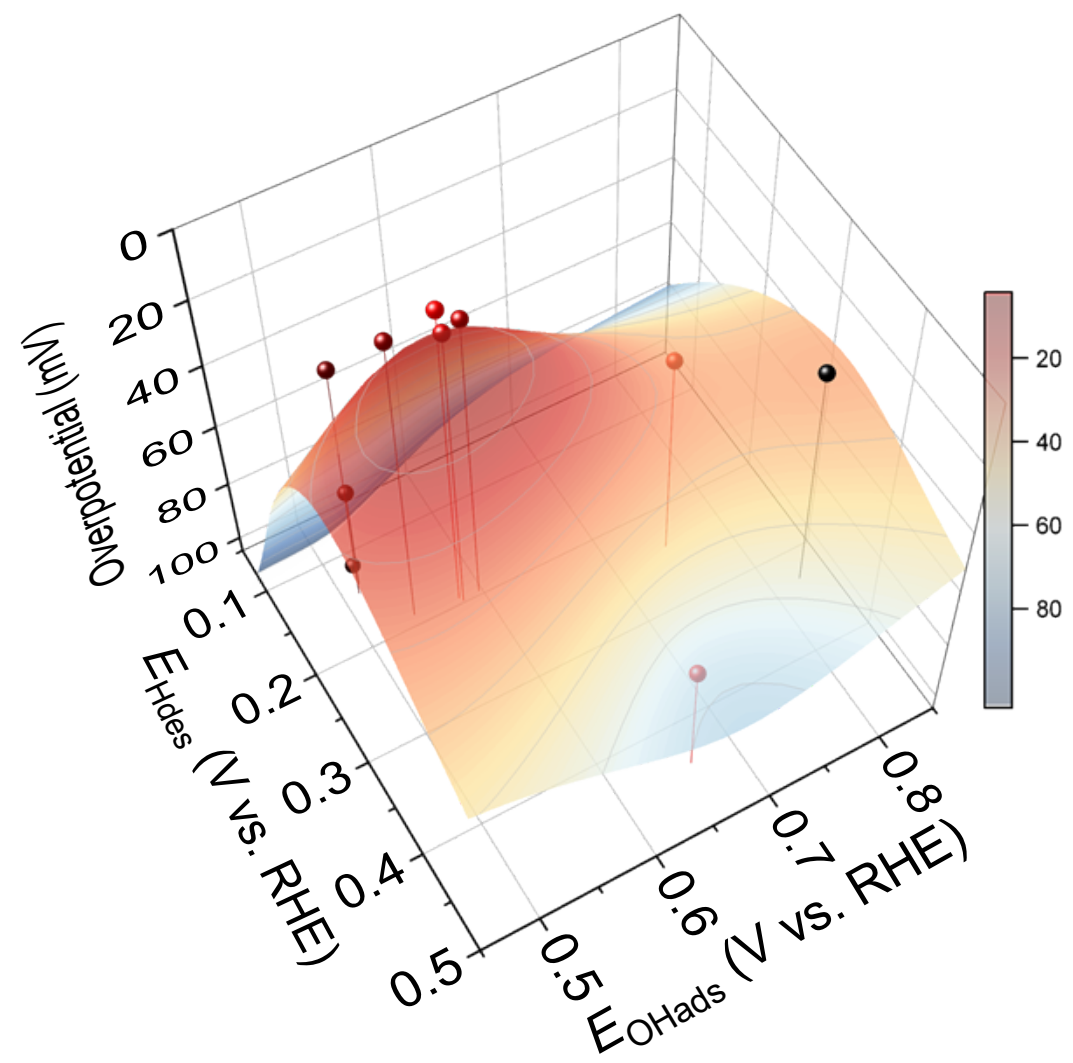

Fig. S17. Experimental 3D activity–HBE–OHBE descriptor map for the RuSi catalyst series, with all raw experimental data points explicitly marked. The interpolated surface is displayed with partial transparency to facilitate visualization of the positions of the measured points relative to the fitted surface. Owing to perspective projection in the 3D rendering, some data points may appear visually displaced from the surface in the two-dimensional view; however, their actual coordinates lie close to the interpolated surface. The interpolated surface is intended only as a visual guide to illustrate the cooperative dependence of HER activity on E_{Hdes} and E_{OHads}, rather than as a quantitatively exact representation of a universal activity surface.

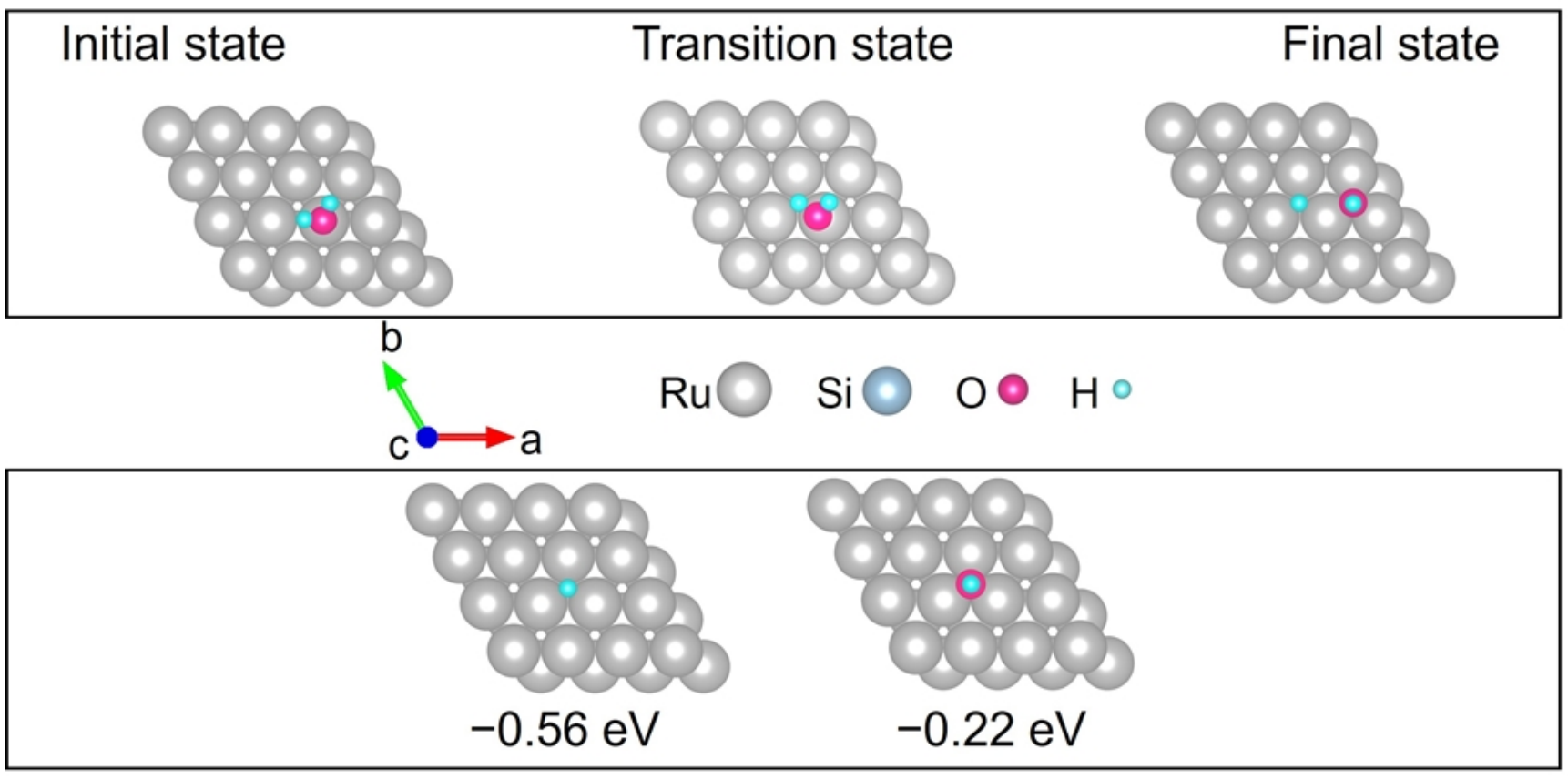

Fig. S18. Water dissociation process on c-Ru with corresponding adsorption configurations of H_2O, H, and OH (upper panel). Most stable adsorption configurations and energies for H and OH on c-Ru (lower panel).

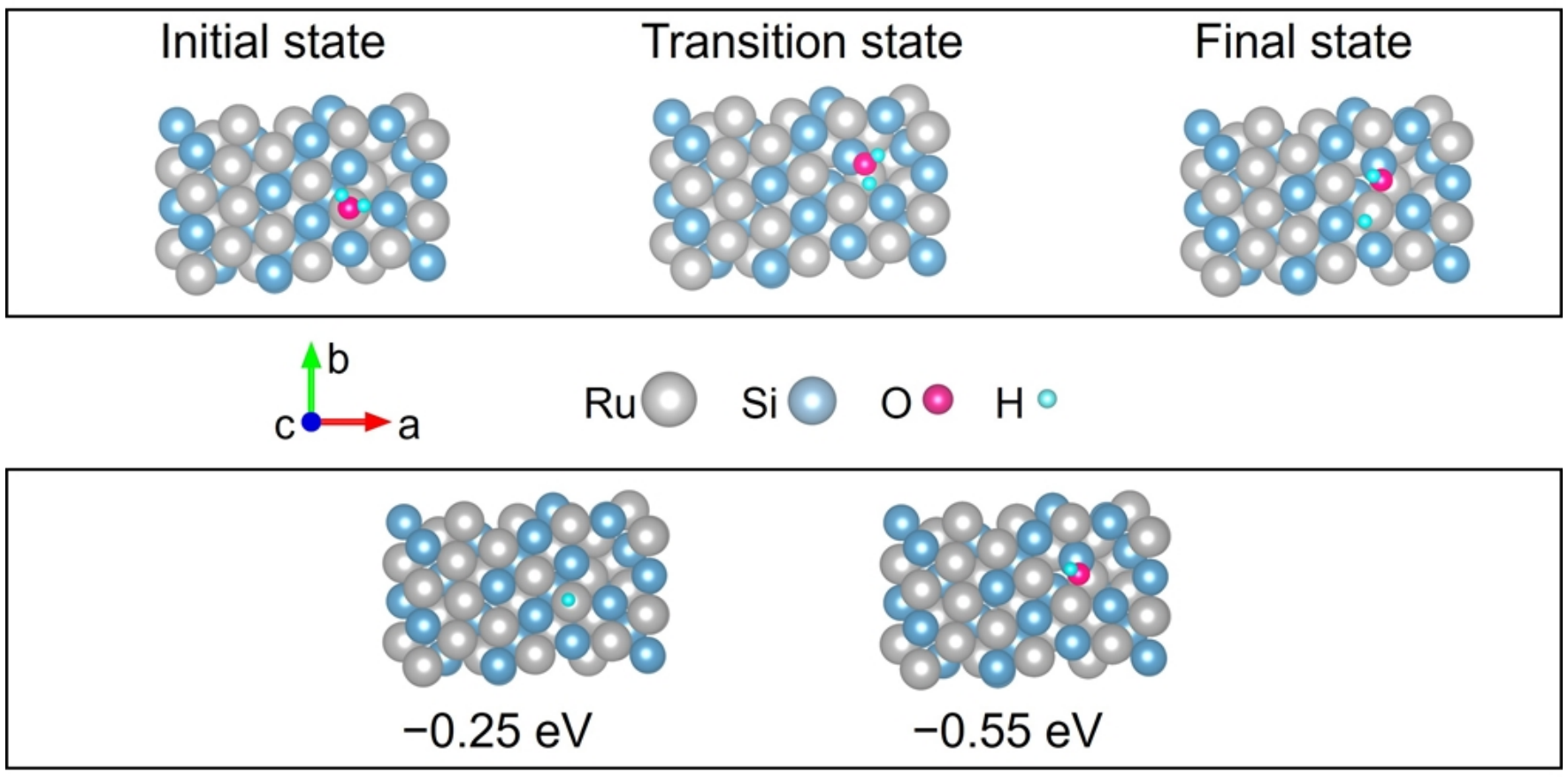

Fig. S19. Water dissociation process on c-RuSi with corresponding adsorption configurations of H_2O, H, and OH (upper panel). Most stable adsorption configurations and energies for H and OH on c-RuSi (lower panel).

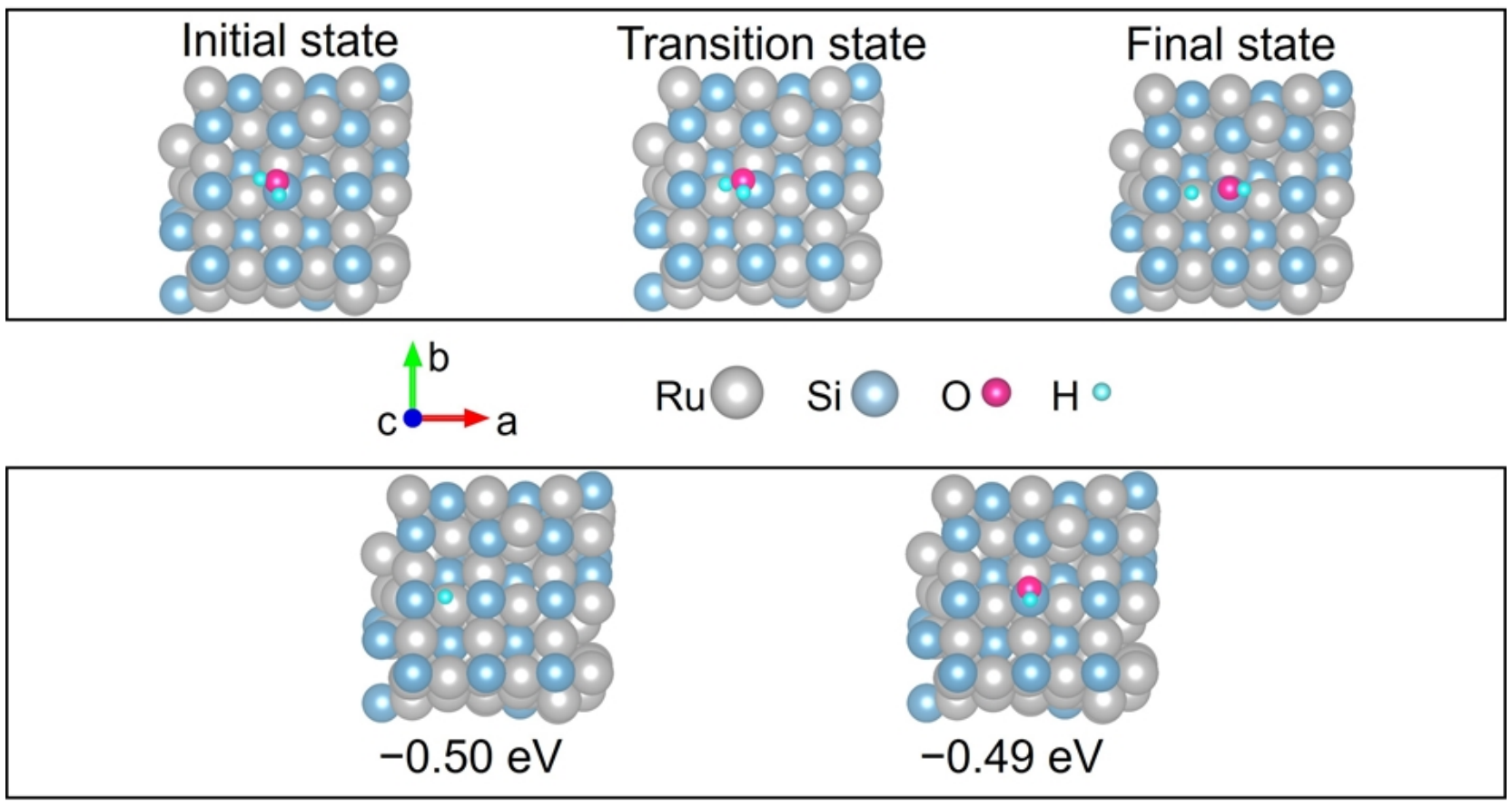

Fig. S20. Water dissociation process on a-RuSi with corresponding adsorption configurations of H_2O, H, and OH (upper panel). Most stable adsorption configurations and energies for H and OH on a-RuSi (lower panel).

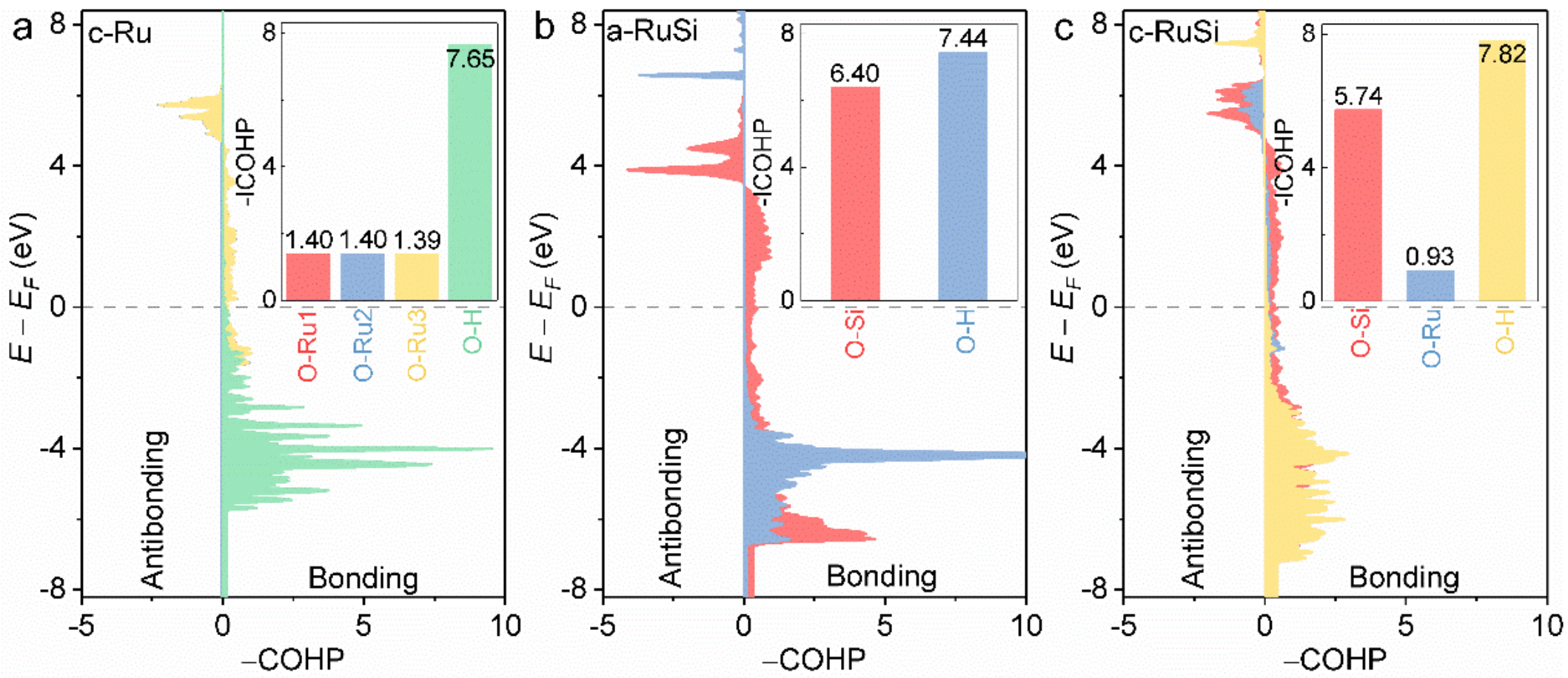

Fig. S21. Crystal orbital hamilton population (COHP) analyses of the optimized OH* adsorption configurations on (a) c-Ru, (b) a-RuSi and (c) c-RuSi. The values shown in the insets are −ICOHP.

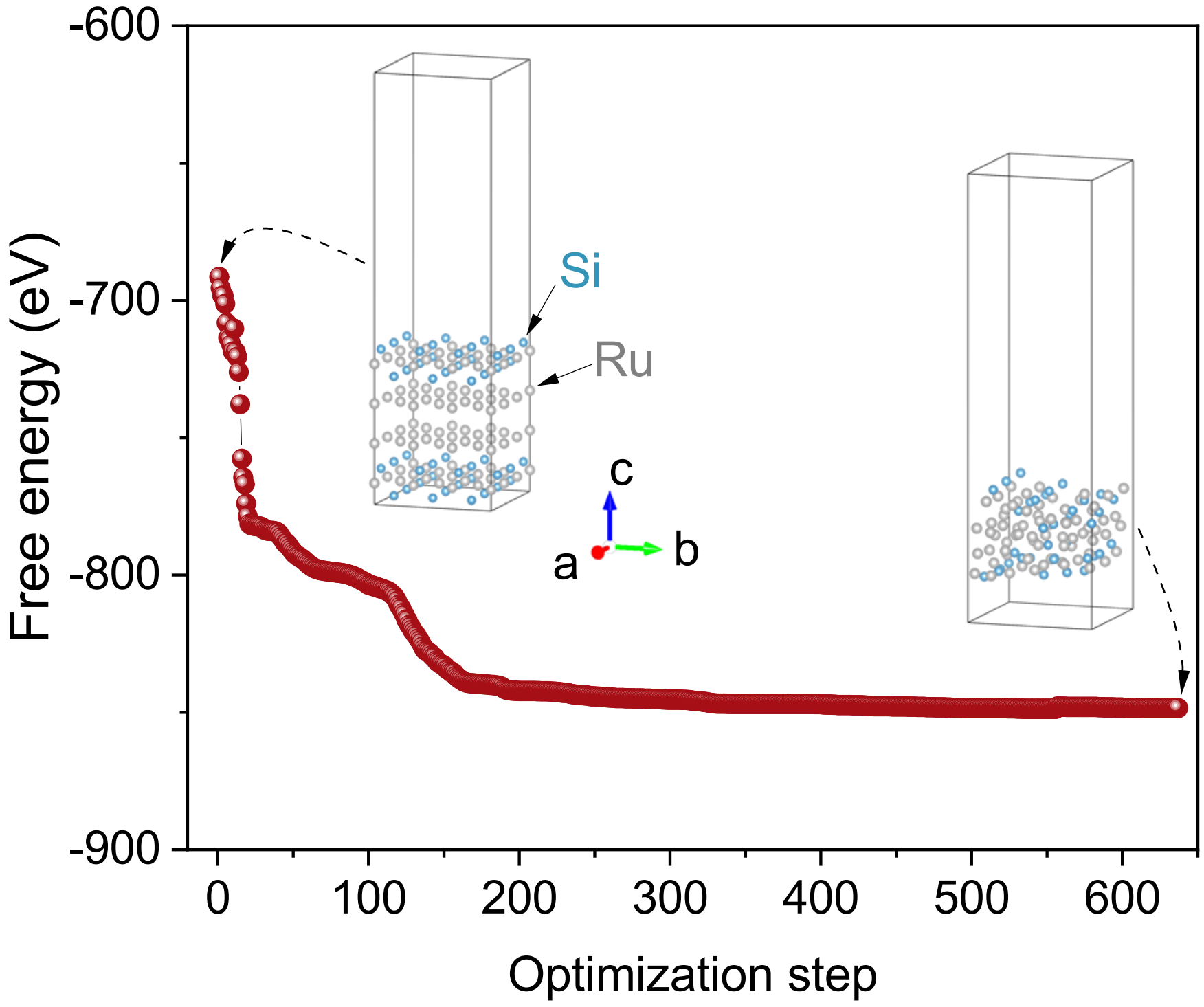

Fig. S22. Structural optimization of the representative disordered a-RuSi model.

Table S1. Activities of various catalysts on glassy carbon in 1 M KOH.

Catalyst	Overpotential at 10 mV cm^{-2} (mV)	Tafel slope (mV/dec)	Reference
a-RuSi	7.85±0.35	29.3±0.42	This work
200 °C	13.8±0.71	32.5±0.71	This work
300 °C	12.85±0.35	32.15±3.04	This work
400 °C	11.75±0.78	29.2±3.11	This work
500 °C	27.15±0.49	45.7±2.40	This work
600 °C	70.9±4.24	83.25±6.01	This work
700 °C	103±7.07	116.75±10.96	This work
PtFeCoNiCuHEA	10.8	28.1	*Nat Commun* **15**, (2024).[10]
Ru/Mo_2C/MoO_2	19	18.3	*Adv Mater* **36**, (2024).[11]
UP-RuNiSAs/C	9	37.6	*Nat Commun* **15**, (2024).[12]
Ru–CrO_x@CN	7.0	30.1	*Nat Catal* **7**, 441-451 (2024).[13]
PtRu/CNT@CeO_{2-x}	17	38	*J Am Chem Soc* **146**, 21453-21465 (2024).[14]
Pt-Ru/RuO_2	18	18.5	*Nat Commun* **15**, (2024).[15]
$Ti_3C_2T_x$-PtSA	38	45	*Nano Lett* **22**, 1398-1405 (2022).[16]
SANi-PtNWs	70	60.3	*Nat Catal* **2**, 495-503 (2019).[17]
Pt@DG	37	53	*J Am Chem Soc* **144**, 2171-2178 (2022).[18]
Pt/np-$Co_{0.85}$Se	58	39	*Nat Commun* **10**, 1743 (2019).[19]
PtSA-NiSe-V	45	52	*Angew Chem, Int Ed* **62**, e202308686 (2023).[20]

Pt1/NMHCS	40	56	*Adv Mater* **33**, e2008599 (2021). [21]
Pt/C60-2	25	55	*Nat Commun* **14**, (2023). [22]
Pt1@Fe-N-C	60	42	*Adv Energy Mater* **8**, (2017). [23]
Pt-Ni(OH)$_x$	58	84	*Adv Energy Mater* **13**, (2023). [24]
Pt1/Mn_3O_4	24	54	*Energy Environ Sci* **15**, 4592-4600 (2022). [25]
Pt-AC/Cr−N−C	19	30	*J Am Chem Soc* **145**, 21432-21441 (2023). [26]
$Ru_{0.3}SrTi_{0.7}O_{3-\delta}$	46	40	*Nat Commun* **11**, 5657 (2020). [27]
Ru SAs/N-Mo_2C	43	38.6	*Applied Catalysis B: Environmental* **277**, (2020). [28]
V_O-Ru/HfO_2-OP	39	29	*Nat Commun* **13**, 1270 (2022). [29]
Ru SAs-MoO_{3-x}/NF	57	51.9	*Adv Sci (Weinh)* **10**, e2300342 (2023). [30]
Ru-$NiCo_2S_4$	32	41.3	*Adv Funct Mater* **32**, (2021). [31]
Ru1CoP/CDs	51	73.4	*Angew Chem, Int Ed* **60**, 7234-7244 (2021). [32]
Ru/np-MoS_2	30	31	*Nat Commun* **12**, 1687 (2021).[33]
Ru@Ni-MOF	22	40	*Angew Chem, Int Ed* **60**, 22276-22282 (2021). [34]
Ru/Co–N–C	23	27.8	*Adv Mater* **34**, e2110103 (2022). [35]
Ru/NC	25	29	*Adv Mater* **35**, e2301133 (2023). [36]
Ru_1/PNC	16	39.4	*J Am Chem Soc* **147**, 3874-3884 (2025). [37]

$Pt_{T-2.8}$	~ 50	95.4	*Nature* **598**, 76–81 (2021). [38]
3D Pt_{SA}-NiO/Ni	26	27.07	*Nat Commun* **12**, 3783 (2021). [39]
Ni_5P_4-Ru	54	52	*Adv Mater* **32**, 1906972 (2020). [40]
Ru_2P/WO_3@NPC	15	18	*Angew Chem Int Ed* **60**, 4110–4116 (2021). [41]
Ru/OMSNNC	13	40.41	*Adv Mater* **33**, 2006965 (2021). [42]
Ni@Ni_2P-Ru NRs	31	41	*J Am Chem Soc* **140**, 2731–2734 (2018). [43]
Ru@CN	32	53	*Energy Environ Sci* **11**, 800–806 (2018). [44]
Ru_{SA+NP}/DC	18.8	35.8	*Adv Sci* **8**, 2004516 (2021). [45]
a-$RuTe_2$ PNRs	36	36	*Nat Commun* **10**, 5692 (2019). [46]
Ru@C2N	17	38	*Nat Nanotechnol* **12**, 441–446 (2017).[47]
Pt_{SA}-$Co(OH)_2$	29	35.72	*Energy Environ Sci* **13**, 3082–3092 (2020). [48]
IrCo@N-C	45	80	*Adv Mater* **30**, 1705324 (2018). [49]
LaRuSi	72	68	*Angew Chem Int Ed* **61**, e202206460 (2022). [50]
$Pt_{1.5}Rh_{1.5}$Tm alloy	20	17.4	*Small* **n/a**, 2400662 (2024). [51]
PtSi	38	81	*Adv Energy Mater* **12**, 2200293 (2022). [52]
IrSi	38	-	*ACS Catal* **12**, 2623-2631 (2022). [53]
$Ru_{d\,=\,2.4\,Å}$	27	32	*Adv Mater* **36**, 2310699 (2024). [54]

NiCo DASs/N-C	189	72.5	*Adv Funct Mater* **33**, 2210867 (2023). [55]
$Co_{NC\text{-}SA}$/N*–C	194	91.9	*ACS Catal* **12**, 10771-10780 (2022). [56]
MSi, M = Ti, Mn, Fe, Ru, Ni, Pd, Co, and Rh	34 – 54	23.6 – 32.3	*Small* **17**, 2006153 (2021). [57]
Co1/PCN	89	52	*Nat Catal* **2**, 134–141 (2019).[58]
$Mo_2TiC_2T_x$-VMo	250	Not stable	*Nat Catal* **1**, 985–992 (2018). [59]
$CoMoS_x$	~ 210 (5mA/cm^2)	-	*Nat Mater* **15**, 197–203 (2016). [60]
C-MoS_2	45	46	*Nat Commun* **10**, 1217 (2019). [61]
MoS_2/NiCo-LDH	78	76.6	*Joule* **1**, 383–393 (2017). [62]

References

1. G. Kresse and D. Joubert, *Phys. Rev. B*, 1999, **59**, 1758–1775.
2. G. Kresse and J. Furthmüller, *Phys. Rev. B*, 1996, **54**, 11169–11186.
3. J. P. Perdew, K. Burke and M. Ernzerhof, *Phys. Rev. Lett.*, 1996, **77**, 3865–3868.
4. P. E. Blöchl, *Phys. Rev. B*, 1994, **50**, 17953–17979.
5. W. Z. Cai, X. Y. He, T. N. Ye, X. M. Hu, C. L. Liu, M. Sasase, M. Kitano, T. Kamiya, H. Hosono and J. Z. Wu, *Small*, 2024, **20**, 2402357.
6. M. Yu and D. R. Trinkle, *J. Chem. Phys.*, 2011, **134**, 064111.
7. E. Sanville, S. D. Kenny, R. Smith and G. Henkelman, *J. Comput. Chem.*, 2007, **28**, 899–908.
8. J. Durst, A. Siebel, C. Simon, F. Hasché, J. Herranz and H. A. Gasteiger, *Energy Environ. Sci.*, 2014, **7**, 2255–2260.
9. J. K. Nørskov, J. Rossmeisl, A. Logadottir, L. Lindqvist, J. R. Kitchin, T. Bligaard and H. Jónsson, *J. Phys. Chem. B*, 2004, **108**, 17886–17892.
10. Z. W. Chen, J. Li, P. Ou, J. E. Huang, Z. Wen, L. Chen, X. Yao, G. Cai, C. C. Yang, C. V. Singh and Q. Jiang, *Nat. Commun.*, 2024, **15**, 359.
11. L. Hou, C. Li, H. Jang, M. G. Kim, J. Z. Jiang, J. Cho, S. Liu and X. Liu, *Adv. Mater.*, 2024, **36**, 2410039.
12. R. Yao, K. Sun, K. Zhang, Y. Wu, Y. Du, Q. Zhao, G. Liu, C. Chen, Y. Sun and J. Li, *Nat. Commun.*, 2024, **15**, 2218.
13. B. Zhang, J. Wang, G. Liu, C. M. Weiss, D. Liu, Y. Chen, L. Xia, P. Zhou, M. Gao, Y. Liu, J. Chen, Y. Yan, M. Shao, H. Pan and W. Sun, *Nat. Catal.*, 2024, **7**, 441–451.
14. C.-A. Zhou, K. Ma, Z. Zhuang, M. Ran, G. Shu, C. Wang, L. Song, L. Zheng, H. Yue and D. Wang, *J. Am. Chem. Soc.*, 2024, **146**, 21453–21465.
15. Y. Zhu, M. Klingenhof, C. Gao, T. Koketsu, G. Weiser, Y. Pi, S. Liu, L. Sui, J. Hou, J. Li, H. Jiang, L. Xu, W.-H. Huang, C.-W. Pao, M. Yang, Z. Hu, P. Strasser and J. Ma, *Nat. Commun.*, 2024, **15**, 1447.
16. J. Zhang, E. Wang, S. Cui, S. Yang, X. Zou and Y. Gong, *Nano Lett.*, 2022, **22**, 1398–1405.
17. M. Li, K. Duanmu, C. Wan, T. Cheng, L. Zhang, S. Dai, W. Chen, Z. Zhao, P. Li, H. Fei, Y. Zhu, R. Yu, J. Luo, K. Zang, Z. Lin, M. Ding, J. Huang, H. Sun, J. Guo, X. Pan, W. A. Goddard, P. Sautet, Y. Huang and X. Duan, *Nat. Catal.*, 2019, **2**, 495–503.
18. Q. Yang, H. Liu, P. Yuan, Y. Jia, L. Zhuang, H. Zhang, X. Yan, G. Liu, Y. Zhao, J. Liu, S. Wei, L. Song, Q. Wu, B. Ge, L. Zhang, K. Wang, X. Wang, C. R. Chang and X. Yao, *J. Am. Chem. Soc.*, 2022, **144**, 2171–2178.
19. K. Jiang, B. Liu, M. Luo, S. Ning, M. Peng, Y. Zhao, Y. R. Lu, T. S. Chan, F. M. F. de Groot and Y. Tan, *Nat. Commun.*, 2019, **10**, 1743.
20. Z. Chen, X. Li, J. Zhao, S. Zhang, J. Wang, H. Zhang, J. Zhang, Q. Dong, W. Zhang, W. Hu and X. Han, *Angew. Chem. Int. Ed.*, 2023, **62**, e202308686.
21. P. Kuang, Y. Wang, B. Zhu, F. Xia, C. W. Tung, J. Wu, H. M. Chen and J. Yu, *Adv. Mater.*, 2021, **33**, e2008599.
22. R. Zhang, Y. Li, X. Zhou, A. Yu, Q. Huang, T. Xu, L. Zhu, P. Peng, S. Song, L.

Echegoyen and F.-F. Li, *Nat. Commun.*, 2023, **14**, 2460.
23. X. Zeng, J. Shui, X. Liu, Q. Liu, Y. Li, J. Shang, L. Zheng and R. Yu, *Adv. Energy Mater.*, 2017, **8**, 1701345.
24. Q. Li, Q. Zhang, W. Xu, R. Zhao, M. Jiang, Y. Gao, W. Zhong, K. Chen, Y. Chen, X. Li and N. Yang, *Adv. Energy Mater.*, 2023, **13**, 2203955.
25. J. Wei, K. Xiao, Y. Chen, X.-P. Guo, B. Huang and Z.-Q. Liu, *Energy Environ. Sci.*, 2022, **15**, 4592–4600.
26. L. Zeng, Z. Zhao, Q. Huang, C. Zhou, W. Chen, K. Wang, M. Li, F. Lin, H. Luo, Y. Gu, L. Li, S. Zhang, F. Lv, G. Lu, M. Luo and S. Guo, *J. Am. Chem. Soc.*, 2023, **145**, 21432–21441.
27. J. Dai, Y. Zhu, H. A. Tahini, Q. Lin, Y. Chen, D. Guan, C. Zhou, Z. Hu, H. J. Lin, T. S. Chan, C. T. Chen, S. C. Smith, H. Wang, W. Zhou and Z. Shao, *Nat. Commun.*, 2020, **11**, 5657.
28. J. Yu, A. Wang, W. Yu, X. Liu, X. Li, H. Liu, Y. Hu, Y. Wu and W. Zhou, *Appl. Catal. B Environ.*, 2020, **277**, 119236.
29. G. Li, H. Jang, S. Liu, Z. Li, M. G. Kim, Q. Qin, X. Liu and J. Cho, *Nat. Commun.*, 2022, **13**, 1270.
30. D. Feng, P. Wang, R. Qin, W. Shi, L. Gong, J. Zhu, Q. Ma, L. Chen, J. Yu, S. Liu and S. Mu, *Adv. Sci.*, 2023, **10**, e2300342.
31. H. Su, S. Song, Y. Gao, N. Li, Y. Fu, L. Ge, W. Song, J. Liu and T. Ma, *Adv. Funct. Mater.*, 2021, **32**, 2109731.
32. H. Song, M. Wu, Z. Tang, J. S. Tse, B. Yang and S. Lu, *Angew. Chem. Int. Ed.*, 2021, **60**, 7234–7244.
33. K. Jiang, M. Luo, Z. Liu, M. Peng, D. Chen, Y. R. Lu, T. S. Chan, F. M. F. de Groot and Y. Tan, *Nat. Commun.*, 2021, **12**, 1687.
34. L. Deng, F. Hu, M. Ma, S. C. Huang, Y. Xiong, H. Y. Chen, L. Li and S. Peng, *Angew. Chem. Int. Ed.*, 2021, **60**, 22276–22282.
35. C. Rong, X. Shen, Y. Wang, L. Thomsen, T. Zhao, Y. Li, X. Lu, R. Amal and C. Zhao, *Adv. Mater.*, 2022, **34**, e2110103.
36. Y. Zhu, K. Fan, C. S. Hsu, G. Chen, C. Chen, T. Liu, Z. Lin, S. She, L. Li, H. Zhou, Y. Zhu, H. M. Chen and H. Huang, *Adv. Mater.*, 2023, **35**, e2301133.
37. H. Jin, X. Chen, Y. Da, L. Fan, R. Jiang, Y. Xiao, B. Yao, Q. He, Y. Yu and W. Chen, *J. Am. Chem. Soc.*, 2025, **147**, 3874–3884.
38. T. He, W. Wang, F. Shi, X. Yang, X. Li, J. Wu, Y. Yin and M. Jin, *Nature*, 2021, **598**, 76–81.
39. K. L. Zhou, Z. L. Wang, C. B. Han, X. X. Ke, C. H. Wang, Y. H. Jin, Q. Q. Zhang, J. B. Liu, H. Wang and H. Yan, *Nat. Commun.*, 2021, **12**, 3783.
40. Q. He, D. Tian, H. Jiang, D. Cao, S. Wei, D. Liu, P. Song, Y. Lin and L. Song, *Adv. Mater.*, 2020, **32**, 1906972.
41. X. Jiang, H. Jang, S. Liu, Z. Li, M. G. Kim, C. Li, Q. Qin, X. Liu and J. Cho, *Angew. Chem. Int. Ed.*, 2020, **60**, 4110–4116.
42. Y. L. Wu, X. Li, Y. S. Wei, Z. Fu, W. Wei, X. T. Wu, Q. L. Zhu and Q. Xu, *Adv. Mater.*, 2021, **33**, 2006965.
43. Y. Liu, S. Liu, Y. Wang, Q. Zhang, L. Gu, S. Zhao, D. Xu, Y. Li, J. Bao and Z.

Dai, *J. Am. Chem. Soc.*, 2018, **140**, 2731–2734.
44. J. Wang, Z. Wei, S. Mao, H. Li and Y. Wang, *Energy Environ. Sci.*, 2018, **11**, 800–806.
45. L. Zhang, H. Jang, Y. Wang, Z. Li, W. Zhang, M. G. Kim, D. Yang, S. Liu, X. Liu and J. Cho, *Adv. Sci.*, 2021, **8**, 2004516.
46. J. Wang, L. Han, B. Huang, Q. Shao, H. L. Xin and X. Huang, *Nat. Commun.*, 2019, **10**, 5692.
47. J. Mahmood, F. Li, S.-M. Jung, M. S. Okyay, I. Ahmad, S.-J. Kim, N. Park, H. Y. Jeong and J.-B. Baek, *Nat. Nanotechnol.*, 2017, **12**, 441–446.
48. K. L. Zhou, C. Wang, Z. Wang, C. B. Han, Q. Zhang, X. Ke, J. Liu and H. Wang, *Energy Environ. Sci.*, 2020, **13**, 308–3092.
49. P. Jiang, J. Chen, C. Wang, K. Yang, S. Gong, S. Liu, Z. Lin, M. Li, G. Xia, Y. Yang, J. Su and Q. Chen, *Adv. Mater.*, 2018, **30**, 1705324.
50. S. Shen, Z. Hu, H. Zhang, K. Song, Z. Wang, Z. Lin, Q. Zhang, L. Gu and W. Zhong, *Angew. Chem. Int. Ed.*, 2022, **61**, e202206460.
51. Q. Li, B. Zhang, C. Sun, X. Sun, Z. Li, Y. Du, J. C. Liu and F. Luo, *Small*, 2024, **20**, 2400662.
52. Z. Pu, T. Liu, G. Zhang, Z. Chen, D. S. Li, N. Chen, W. Chen, Z. Chen and S. Sun, *Adv. Energy Mater.*, 2022, **12**, 2200293.
53. D. Chen, Z. Pu, P. Wang, R. Lu, W. Zeng, D. Wu, Y. Yao, J. Zhu, J. Yu, P. Ji and S. Mu, *ACS Catal.*, 2022, **12**, 2623–2631.
54. B. Jiang, J. Zhu, Z. Xia, J. Lyu, X. Li, L. Zheng, C. Chen, S. Chaemchuen, T. Bu, F. Verpoort, S. Mu, J. Wu, J. Wang and Z. Kou, *Adv. Mater.*, 2023, **36**, 2310699.
55. M. Li, H. Zhu, Q. Yuan, T. Li, M. Wang, P. Zhang, Y. Zhao, D. Qin, W. Guo, B. Liu, X. Yang, Y. Liu and Y. Pan, *Adv. Funct. Mater.*, 2022, **33**, 2210867.
56. M. Wang, K. Sun, W. Mi, C. Feng, Z. Guan, Y. Liu and Y. Pan, *ACS Catal.*, 2022, **12**, 10771–10780.
57. Y. He, T. L. Wang, M. Zhang, T. W. Wang, L. F. Wu, L. Zeng, X. Wang, M. Boubeche, S. Wang, K. Yan, S. H. Lin and H. Luo, *Small*, 2021, **17**, 2006153.
58. L. Cao, Q. Luo, W. Liu, Y. Lin, X. Liu, Y. Cao, W. Zhang, Y. Wu, J. Yang, T. Yao and S. Wei, *Nat. Catal.*, 2018, **2**, 134–141.
59. J. Zhang, Y. Zhao, X. Guo, C. Chen, C.-L. Dong, R.-S. Liu, C.-P. Han, Y. Li, Y. Gogotsi and G. Wang, *Nat. Catal.*, 2018, **1**, 985–992.
60. J. Staszak-Jirkovský, Christos D. Malliakas, Pietro P. Lopes, N. Danilovic, Subrahmanyam S. Kota, K.-C. Chang, B. Genorio, D. Strmcnik, Vojislav R. Stamenkovic, M. G. Kanatzidis and N. M. Markovic, *Nat. Mater.*, 2015, **15**, 197–203.
61. Y. Zang, S. Niu, Y. Wu, X. Zheng, J. Cai, J. Ye, Y. Xie, Y. Liu, J. Zhou, J. Zhu, X. Liu, G. Wang and Y. Qian, *Nat. Commun.*, 2019, **10**, 1217.
62. J. Hu, C. Zhang, L. Jiang, H. Lin, Y. An, D. Zhou, M. K. H. Leung and S. Yang, *Joule*, 2017, **1**, 383–393.
